# Integrative analysis of *KRAS* wildtype metastatic pancreatic ductal adenocarcinoma reveals mutation and expression-based similarities to cholangiocarcinoma

**DOI:** 10.1038/s41467-022-33718-7

**Published:** 2022-10-08

**Authors:** James T. Topham, Erica S. Tsang, Joanna M. Karasinska, Andrew Metcalfe, Hassan Ali, Steve E. Kalloger, Veronika Csizmok, Laura M. Williamson, Emma Titmuss, Karina Nielsen, Gian Luca Negri, Sandra E. Spencer Miko, Gun Ho Jang, Robert E. Denroche, Hui-li Wong, Grainne M. O’Kane, Richard A. Moore, Andrew J. Mungall, Jonathan M. Loree, Faiyaz Notta, Julie M. Wilson, Oliver F. Bathe, Patricia A. Tang, Rachel Goodwin, Gregg B. Morin, Jennifer J. Knox, Steven Gallinger, Janessa Laskin, Marco A. Marra, Steven J. M. Jones, David F. Schaeffer, Daniel J. Renouf

**Affiliations:** 1 Pancreas Centre BC, Vancouver, BC Canada; 2 Division of Medical Oncology, BC Cancer, Vancouver, BC Canada; 3 Department of Pathology and Laboratory Medicine, UBC, Vancouver, BC Canada; 4 Canada’s Michael Smith Genome Sciences Centre, BC Cancer, Vancouver, BC Canada; 5 Ontario Institute for Cancer Research, Toronto, ON Canada; 6 Departments of Surgery and Oncology, Cummings School of Medicine, University of Calgary, Calgary, AB Canada; 7 The Ottawa Hospital Cancer Centre, Ottawa Hospital Research Institute, Ottawa, ON Canada; 8 Department of Medical Genetics, University of British Columbia, Vancouver, BC Canada; 9 University Health Network, University of Toronto, Toronto, ON Canada; 10 Division of Anatomic Pathology, Vancouver General Hospital, Vancouver, BC Canada; 11 Department of Medicine, University of British Columbia, Vancouver, BC Canada

**Keywords:** Cancer genomics, Pancreatic cancer, Cancer genomics, Cellular signalling networks, Cancer genomics

## Abstract

Oncogenic *KRAS* mutations are absent in approximately 10% of patients with metastatic pancreatic ductal adenocarcinoma (mPDAC) and may represent a subgroup of mPDAC with therapeutic options beyond standard-of-care cytotoxic chemotherapy. While distinct gene fusions have been implicated in *KRAS* wildtype mPDAC, information regarding other types of mutations remain limited, and gene expression patterns associated with *KRAS* wildtype mPDAC have not been reported. Here, we leverage sequencing data from the PanGen trial to perform comprehensive characterization of the molecular landscape of *KRAS* wildtype mPDAC and reveal increased frequency of chr1q amplification encompassing transcription factors *PROX1* and *NR5A2*. By leveraging data from colorectal adenocarcinoma and cholangiocarcinoma samples, we highlight similarities between cholangiocarcinoma and *KRAS* wildtype mPDAC involving both mutation and expression-based signatures and validate these findings using an independent dataset. These data further establish *KRAS* wildtype mPDAC as a unique molecular entity, with therapeutic opportunities extending beyond gene fusion events.

## Abstract (web summary)

*KRAS* wildtype metastatic pancreatic ductal adenocarcinoma (mPDAC) could represent a distinct molecular entity from other PDACs. Here, the authors analyse *KRAS* wildtype mPDAC tumours using genomics and transcriptomics and find molecular similarities with cholangiocarcinomas.

## Introduction

Pancreatic ductal adenocarcinoma (PDAC) is projected to become the second most common cause of cancer-related deaths in the United States by 2030^1^. Oncogenic driver mutations in *KRAS* are a hallmark genomic event in PDAC and occur in approximately 90% of patients, a prevalence that is much higher compared to other cancers^2^. Mutant KRAS drives tumor progression by activating downstream cell proliferation pathways, immunosuppression, and cell metabolism reprogramming, which in combination with loss of function alterations in tumor suppressor genes such as *TP53*, *SMAD4*, and *CDKN2A*, fuels PDAC tumor growth and metastasis^3,4^. While efforts to pharmacologically target oncogenic KRAS have intensified recently, most variants remain resistant to targeted approaches. Meanwhile, there is a growing interest toward understanding the underlying biology of *KRAS* wildtype PDAC tumors, which are unique in that they often also lack secondary mutations in tumor suppressor genes commonly found in PDAC^5^.

Next-generation sequencing (NGS) has facilitated the genomic characterization of several PDAC cohorts which have included *KRAS* wildtype tumors^6–9^. We and others have identified oncogenic fusions involving *NRG1* in *KRAS* wildtype metastatic PDAC (mPDAC) and showed that such tumors are sensitive to the ERBB inhibitor afatinib^5,10^. While such studies have characterized the distinct fusion landscape observed in *KRAS* wildtype mPDAC, transcriptome-based differences between *KRAS* wildtype and mutant mPDAC remain unexplored. Gene expression-based molecular subtypes of PDAC have been broadly classified into basal-like and classical subgroups, with basal-like tumors being associated with poor prognosis and resistance to first-line chemotherapy^11,12^. As studies interrogating the genomic landscape of *KRAS* wildtype mPDAC have indicated that these tumors may represent a distinct molecular entity, transcriptome-based profiling is needed to better understand how *KRAS* wildtype tumors align to the molecular subtypes of PDAC, as well as identify other potential therapeutic opportunities.

Here, we perform comprehensive genome and transcriptome characterization of *KRAS* wildtype mPDAC using data from patients enrolled in the PanGen trial of mPDAC (NCT02869802). By leveraging additional sequencing data from metastatic colorectal adenocarcinoma and cholangiocarcinoma cancer types as well as two independent cohorts of mPDAC samples, we highlight results indicative of roles of transcription factors *NR5A2* and *PROX1* in *KRAS* wildtype mPDAC as well as a distinct similarity between cholangiocarcinoma and *KRAS* wildtype mPDAC samples.

## Results

### Clinical characterization of KRAS wildtype mPDAC in the PanGen cohort

A total of 63 patients with newly diagnosed mPDAC were enrolled and received sequencing as part of the PanGen trial (NCT02869802), prior to receiving treatment for their metastatic disease, between October 2016 and May 2021. Nine of 63 (14%) patients had *KRAS* wildtype tumors by whole-genome sequencing (WGS) and were investigated for differences in clinical attributes collected as part of the trial (Supplementary Table 1). Patients with *KRAS* wildtype tumors were diagnosed earlier on average at 51.4 years (IQR 48.8–55.2) compared to 60.9 years (IQR 56.7–67.4) in the *KRAS* mutant group (*p* = 0.03). CA19-9 levels in the *KRAS* wildtype group were lower compared to the *KRAS* mutant group (median 58 vs. 4900 U/mL in the *KRAS* mutant group; *p* = 0.03). Other clinical features including history of diabetes, family history of malignancy, and tumor grade were comparable between the two groups. Approximately half of all patients in the study received FOLFIRINOX as first-line therapy (56 and 52% of *KRAS* wildtype and mutant tumors, respectively). One of nine (11%) and 10 of 54 (19%) *KRAS* wildtype and mutant groups (respectively) received first-line immunotherapy as part of a phase II randomized clinical trial with an intervention arm consisting of front-line gemcitabine, nab-paclitaxel, durvalumab, and tremelimumab (NCT02879318). All but one patient in the *KRAS* wildtype group presented with de novo metastatic disease. One patient with an *NTRK2* fusion developed recurrent disease after total pancreatectomy and adjuvant FOLFIRINOX, and remained on first-line gemcitabine/nab-paclitaxel. One patient in the *KRAS* mutant group received second-line palbociclib for a *CDKN2A* mutation, as part of a clinical trial (NCT03297606).

**Fig. 1:**
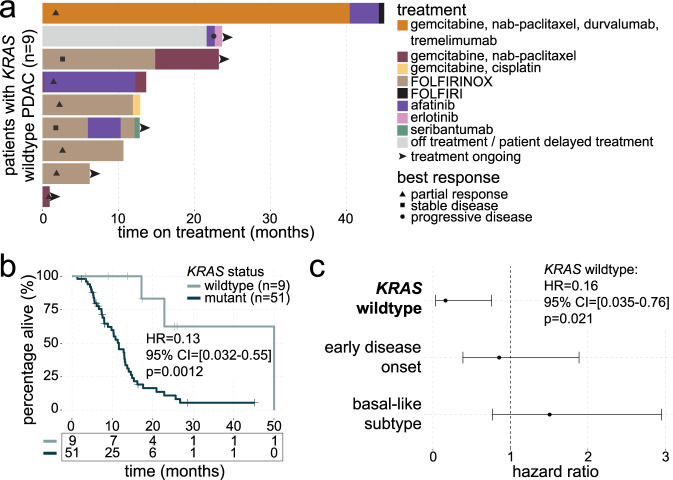
Administration of targeted treatment regimens in patients with *KRAS* wildtype mPDAC. **a** Swimmer’s plot showing the duration of each treatment regimen received by each patient with *KRAS* wildtype mPDAC (*n* = 9). Best response to first-line therapy is indicated by black symbols. Black arrows at the end of bars indicate patients who remain on treatment. **b** Kaplan–Meier curve comparing overall survival between *KRAS* wildtype (*n* = 9) and mutant (*n* = 51) groups. Hazard ratio (HR), 95% confidence interval (CI), and log-rank *p* value are shown. **c** Forest plot showing results of multivariate survival analysis (*n* = 60). Significance of *KRAS* wildtype status (*p* value) shown is based on the Wald statistic. For each covariate, dots represent the hazard ratio and lines represent 95% confidence intervals. Source data are provided as a Source Data file.

Molecular-targeted therapies were received by four of nine (44%) patients with *KRAS* wildtype mPDAC, which included afatinib (four patients) and post-afatinib administration of erlotinib (one patient; Fig. 1a), and were administered to patients based on an oncogenic fusion detected in their tumor. Three patients with *KRAS* mutant tumors that were enrolled in clinical trials for which primary results have not been published (NCT03297606, one patient; NCT03450018, two patients) were excluded from overall survival (OS) analysis. Patients with *KRAS* wildtype mPDAC showed increased OS in univariate analysis (hazard ratio (HR) = 0.13, 95% confidence interval (CI) = [0.032–0.55], log-rank *p* = 0.0012; Fig. 1b), and the prognostic effect of *KRAS* mutation status remained significant (HR = 0.16, 95% CI = [0.035–0.76], *p* = 0.021) when multivariate survival analysis was performed, which included age of disease onset (≤55 vs. >55 years^13^) and Moffitt subtype as these variables were deemed significant (*p* < 0.1) in a Forward Selection analysis of covariates (Fig. 1c). To further explore the association between *KRAS* mutation status and survival in PDAC, we leveraged sequencing data from the COMPASS (mPDAC; *n* = 195), and Hartwig (mPDAC; *n* = 113) cohorts. Genome sequencing data revealed *KRAS* wildtype status in 18/195 (9%) and 16/113 (14%) COMPASS and Hartwig samples, respectively. While OS was not significantly different between patients with *KRAS* wildtype and mutant tumors in the COMPASS cohort (HR = 1.1, 95% CI = [0.62–1.8], *p* = 0.81), patients with *KRAS* wildtype tumors showed significantly higher OS in the Hartwig cohort (HR = 0.25, 95% CI = [0.088–0.73], *p* = 0.0070; Supplementary Fig. 1). Interestingly, fusion rates among *KRAS* wildtype tumors were higher in the Hartwig cohort (five of 16 (31%) patients; *BRAF-NRF1* (two patients, fusion partners *NRF1* and *SND1*), *ALK-EML4* (one patient), *NTRK3-EML4* (one patient), and *FGFR2-KIAA1598* (one patient)) compared to COMPASS (one of 18 (6%) patients; *NTRK3-EML4*), suggesting that underlying cohort-specific genomic differences may explain the survival discrepancies between mPDAC cohorts, though this extends outside the scope of the current study.

### Somatic mutation landscape of *KRAS* wildtype mPDAC

**Fig. 2:**
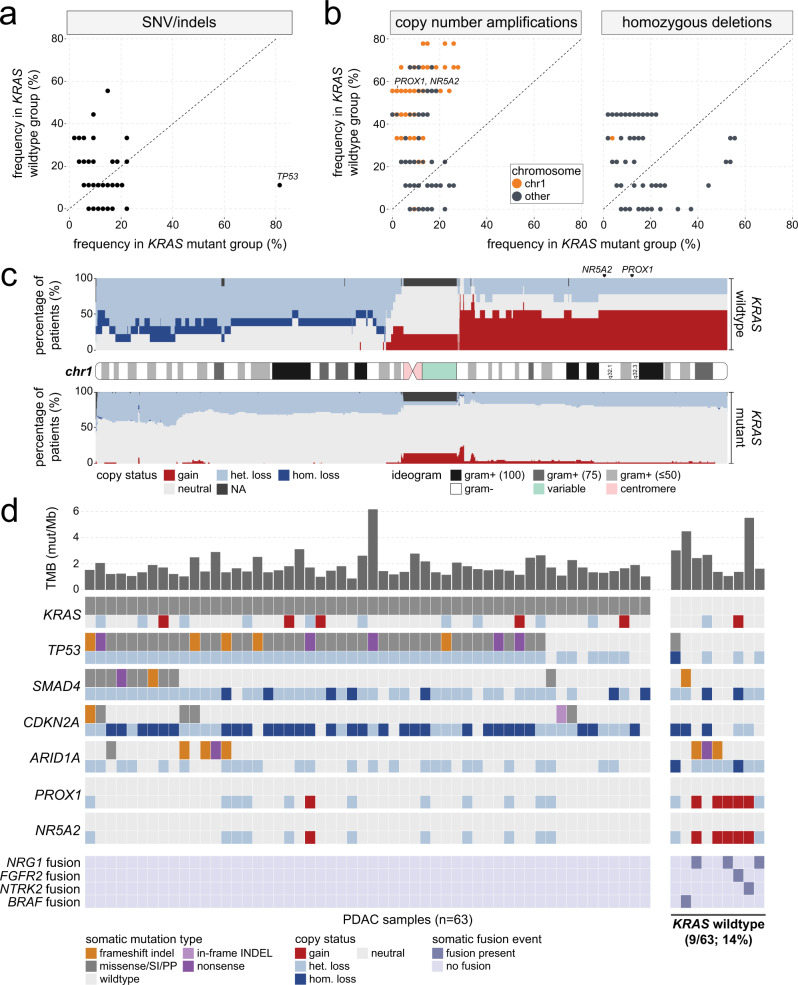
Patients with *KRAS* wildtype mPDAC harbor distinct fusion events that enable targeted therapeutic approaches. **a** Scatter plot comparing SNV/indel frequency across genes in *KRAS* wildtype versus mutant groups. **b** Scatter plot comparing copy number amplification (left) and deletion (right) events across genes in *KRAS* wildtype versus mutant groups. Colors indicate whether the gene is located on chromosome 1 (chr1). **c** Stacked bar plot showing CNV frequency across the length of chr1 in *KRAS* wildtype (upper) and mutant (lower) samples. The corresponding ideogram for chr1 is displayed between the *KRAS* wildtype and mutant tracks and is colored based on reported gram-staining patterns. **d** Oncoprint summarizing somatic SNV/indel, CNV, and fusion landscape in metastatic PDAC tumors. For each gene, CNV tracks are shown immediately below each SNV track. Fusion genes are shown in the bottom-most four tracks. *KRAS* wildtype mPDAC tumors are shown on the far right. Upper bars represent tumor mutational burden (TMB) levels. Source data are provided as a Source Data file.

To characterize the somatic mutation landscape of *KRAS* wildtype samples in the PanGen cohort, we performed an exploratory analysis in which the frequency of single nucleotide variant (SNV)/indel and copy number amplification/deletion events were compared for each gene between *KRAS* wildtype (*n* = 9) and mutant (*n* = 54) groups. SNV/indels affecting *TP53* were less frequent in *KRAS* wildtype (11.1%) compared to mutant (81.5%) tumors (Fig. 2a; *p* = 0.010), while no other genes showed significant differences in SNV/indel frequency between groups. Higher frequency of copy number amplification was observed for 1395 genes on chromosome 1 (chr1; Supplementary Data 1), including transcription factors *NR5A2* and *PROX1* (Fig. 2b). *NR5A2*, also known as *LRH-1*, is an essential regulator of gene transcription programs including cholesterol homeostasis^14^ and has been linked to pancreatic cancer susceptibility^15^, development and progression^16^. *PROX1* is a homeobox transcription factor with critical roles in embryogenesis and cell fate determination and has been linked to both oncogenic and tumor suppressor roles in cancer^17^, while increased PROX1 levels have been associated with improved survival in patients with PDAC^18^, suggesting that PROX1 may have opposing roles that are dependent on tumor type or disease stage. Besides chr1, we also noted a higher frequency of copy number amplifications for 350 genes on chromosome 8 (chr8). No genes showed significant differences in frequency of homozygous deletion between *KRAS* wildtype and mutant groups.

To visualize the spatial distribution of copy number variation (CNV) events, we mapped the copy status of *KRAS* wildtype and mutant samples to 100 kb bins along the entire length of chr1, which revealed broad amplification of the entire q arm (chr1q) in approximately 50% of *KRAS* wildtype tumors (Fig. 2c). Analysis of chr8 showed a copy amplification pattern that was unique to the *KRAS* wildtype group, in which broad amplification of one chromosomal arm (chr8q) was observed in approximately 33% of patients (Supplementary Fig. 2). We next sought to validate our findings of *KRAS* wildtype-associated SNV/indel and CNVs in the validation PDAC cohorts. Similar to the PanGen cohort, SNV/indel frequency in *TP53* was lower in *KRAS* wildtype tumors in both COMPASS (*p* = 6.3e–5) and Hartwig (*p* = 0.0054) cohorts (Supplementary Fig. 3a). Meanwhile, neither *NR5A2* nor *PROX1* amplification rates were higher in *KRAS* wildtype tumors belonging to either validation cohort (Supplementary Fig. 3b), indicating that chr1q amplification is unique to *KRAS* wildtype tumors in the PanGen cohort.

We next assessed the combinatory occurrence of SNV, CNV, and fusion events in each sample (Fig. 2d). Oncogenic somatic fusions were identified in six of nine (66.7%) *KRAS* wildtype tumors, and these fusions were not detected in any *KRAS* mutant tumors (*p* = 1.2e–6). Fusions identified in *KRAS* wildtype tumors included *NRG1* (fusion partners *ATP1B1* (two patients) and *APP* (one patient)), *FGFR2-GCC2* (one patient), *NTRK2-THAP1* (one patient), and *BRAF-TNS3* (one patient), and were likely to be bioactive events given that each fusion resulted in an in-frame transcript and was assigned a high-quality threshold by the Arriba fusion caller^19^. Tumor mutational burden (TMB) levels were not significantly different between *KRAS* wildtype (median TMB = 2.4 mut/Mb) and mutant (median TMB = 1.5 mut/Mb) groups (*p* = 0.11).

### Transcriptional landscape of wildtype PDAC

**Fig. 3:**
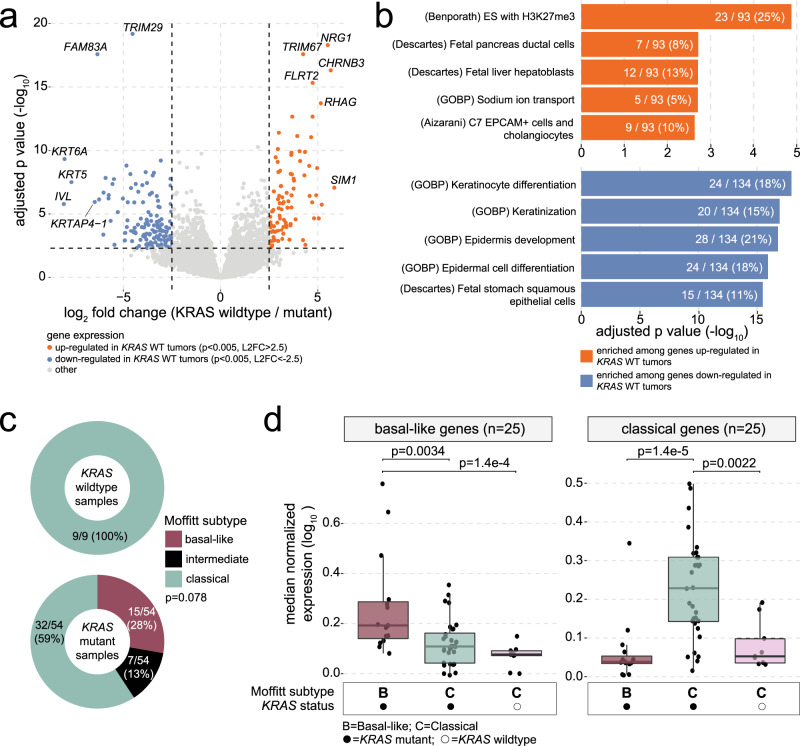
Differential expression analysis reveals significantly higher mRNA expression of cholangiocyte-associated genes in *KRAS* wildtype tumors. **a** Volcano plot showing mRNA-based differential expression analysis (DEA; based on two-tailed Wald tests followed by Benjamini–Hochberg multiple test correction) results between *KRAS* wildtype (*n* = 9) and mutant (*n* = 54) tumors. Each point represents a gene, and genes are colored based on up (orange) and down (blue) regulation in *KRAS* wildtype versus mutant groups. **b** Bar plots demonstrating results of enrichment analysis (one-tailed hypergeometric tests; Benjamini–Hochberg multiple test correction) performed on up (orange; upper) and down (blue; lower) regulated genes. Genes upregulated in *KRAS* wildtype tumors are significantly enriched for genes known to be uniquely expressed in cholangiocytes. **c** Donut plots showing the distribution of Moffitt subtype calls across *KRAS* wildtype (top) and mutant (bottom) groups. Two-tailed Fisher’s exact test *p* value shown. **d** Box plots comparing median mRNA expression levels of Moffitt basal-like (left) and classical (right) genes across *KRAS* wildtype and mutant groups, stratified by Moffitt subtype. Left to right: *KRAS* mutant basal-like samples (*n* = 15), *KRAS* mutant classical samples (*n* = 32), *KRAS* wildtype classical samples (*n* = 9). Box plots indicate median (central line), 25–75% IQR (bounds of box), and whiskers extend from box bounds to the largest value no further than 1.5 times the IQR. Two-tailed Wilcoxon mean rank-sum *p* values are shown. Source data are provided as a Source Data file.

To characterize the transcriptional landscape of *KRAS* wildtype PDAC, we performed differential expression analysis (DEA) between *KRAS* wildtype (*n* = 9) and mutant (*n* = 54) groups (Fig. 3a). DEA revealed significant (*p* < 0.05) up- and downregulation of 1132 and 1265 genes (respectively) in *KRAS* wildtype tumors (Supplementary Data 2), while more conservative significance thresholds (*p* < 0.005, absolute log_2_ fold change (L2FC) > 2.5) were used to define a smaller list of up (*n* = 93) and downregulated (*n* = 134) genes for input to downstream pathway analysis (Supplementary Data 3). This set of 227 conservatively DE genes will henceforth be referred to as the *KRAS* mutation status signature genes. Exploratory enrichment analysis was performed separately on the conservatively up- and downregulated genes and encompassed 32,284 gene sets^20^ (Fig. 3b and Supplementary Data 4 and 5). Genes upregulated in *KRAS* wildtype tumors were enriched for known targets of the repressive epigenetic mark H3K27me3 (*p* = 1.3e–5) as well as genes related to ductal (*p* = 0.0019) and hepatoblast (*p* = 0.0020) cell types. Interestingly, genes known to be more highly expressed in cholangiocytes compared to other liver cell types, as identified by Aizarani et al.^21^, were also enriched among genes upregulated in *KRAS* wildtype tumors (*p* = 0.0023) and included *CTNND2*, *DCDC2*, *FXYD2*, *CFTR*, *SLC4A4*, *WNK2*, *PKHD1*, *CHST9*, and *KCNJ16*. Genes downregulated in *KRAS* wildtype tumors were significantly enriched for cellular differentiation pathways, including keratinocyte (*p* = 1.2e–18) and epidermal (*p* = 1.2e–16) cell types. To ascertain whether *KRAS* wildtype versus mutant differences in gene expression might be confounded by the clinical variables that were significantly associated with *KRAS* mutation status, we calculated the correlation between expression of each of the 227 DE genes with age at diagnosis and median CA19-9 levels at baseline (Supplementary Fig. 4). Of note, zero of the nine upregulated genes that overlapped with the Aizarani et al. cholangiocyte gene set were significantly correlated with either clinical variable. Out of all 227 DE genes, only *CR2* showed a significant correlation (rho = −0.50, *p* = 0.010) with CA19-9 levels, and we therefore concluded that the clinical variables associated with *KRAS* mutation status were unlikely confounding the DEA results. We next assessed *KRAS* wildtype-associated differences in mRNA levels for the DE genes (*p* < 0.05 or *p* < 0.005 and absolute L2FC > 2.5 in PanGen) in each of the validation cohorts. In support of our results in the PanGen cohort, genes downregulated in *KRAS* wildtype samples in PanGen showed significantly (*p* < 0.05) lower L2FC (*KRAS* wildtype/mutant) values in both COMPASS and Hartwig cohorts, while genes upregulated in *KRAS* wildtype samples in PanGen showed significantly higher L2FC values, with the exception of conservative threshold (*p* < 0.005, absolute L2FC > 2.5) genes for the Hartwig dataset (*p* = 0.26; Supplementary Fig. 5). In particular, *FXYD2* and *WNK2*, belonging to the cholangiocyte-specific gene set^21^, showed significantly increased mRNA levels in *KRAS* wildtype samples in the COMPASS (L2FC = 2.9, *p* = 0.0024) and Hartwig (L2FC = 3.1, *p* = 2.5e–7) cohorts, respectively.

### Expression-based subtyping of *KRAS* wildtype PDAC

Previous studies have demonstrated the ability to condense inter-sample heterogeneity of PDAC into discrete molecular subtypes^3,8^, which often converge onto the basal-like (or “squamous”) and classical subtypes proposed by Moffitt et al.^22^ along with an intermediate subtype that may represent a hybrid or transitional state^23,24^. Given the positive relationship between oncogenic *KRAS* gene imbalance and the basal-like subtype signature in PDAC^23–25^, we hypothesized that Moffitt subtyping calls and expression patterns differ according to *KRAS* mutation status. Moffitt subtyping of each sample revealed over-representation of classical-subtype calls among *KRAS* wildtype samples (nine of nine; 100%) compared to *KRAS* mutant samples (32/54; 59%; *p* = 0.021; Fig. 3c). Among the conservative (*p* < 0.005, absolute L2FC > 2.5) list of DE genes, nine basal-like (*FAM83A, KRT6A, CST6, LY6D, SLC2A1, SCEL, DHRS9, SERPINB4*, and *S100A2*) and four classical (*VSIG2, KRT20, TFF2*, and *TFF3*) genes were downregulated in *KRAS* wildtype tumors. *KRAS* wildtype classical samples showed significantly lower median mRNA expression of classical genes when compared to *KRAS* mutant classical samples (*p* = 0.0022; Fig. 3d), indicating that *KRAS* wildtype mPDAC may not conform to the classical subtype of PDAC. Subtyping according to the Collisson method^8^ revealed significantly higher proportion of exocrine-like calls among *KRAS* wildtype (six of nine; 67%) compared to mutant (three of 54; 6%) group (*p* = 1.2e–4; Supplementary Fig. 6a), though median expression of exocrine-like genes remained low across all samples apart from one *KRAS* wildtype sample showing high outlier expression (Supplementary Fig. 6b). Subtyping according to the Bailey method^3^ showed significant overlap between *KRAS* wildtype tumors and the ADEX subtype (*p* = 5.5e–6; Supplementary Fig. 6c), though differences in median expression of ADEX subtyping genes was not significantly different between *KRAS* wildtype samples and any of the *KRAS* mutant subtype groups (Supplementary Fig. 6d). Distribution of metabolic subtype calls was significantly different between *KRAS* wildtype and mutant groups (*p* = 0.023), with the *KRAS* wildtype group showing higher proportion of cholesterogenic calls (44.4% vs. 13.0% in the *KRAS* mutant group) and lower proportion of glycolytic calls (11.1% vs. 42.6% in the *KRAS* mutant group). While median expression of cholesterogenic genes was not significantly different between groups (*p* = 0.99), median expression of glycolytic genes trended toward lower values in the *KRAS* wildtype group (*p* = 0.069).

We next compared Moffitt subtyping patterns between *KRAS* wildtype and mutant groups in the validation cohorts. Frequency of Moffitt subtype calls was not significantly different across *KRAS* wildtype and mutant samples in the COMPASS (*p* = 0.91) nor Hartwig (*p* = 0.44) cohorts (Supplementary Fig. 7a). However, when comparing Moffitt classical *KRAS* mutant samples versus all *KRAS* wildtype samples, we observed significantly lower mRNA expression of classical genes in the *KRAS* wildtype group in the Hartwig cohort (*p* = 9.9e–4; Supplementary Fig. 7b), consistent with the notion that *KRAS* wildtype PDAC samples may not accurately fall into the two canonical Moffitt subtyping groups.

### Effect of *KRAS* wildtype-associated chr1 amplification on mRNA and protein expression levels

**Fig. 4:**
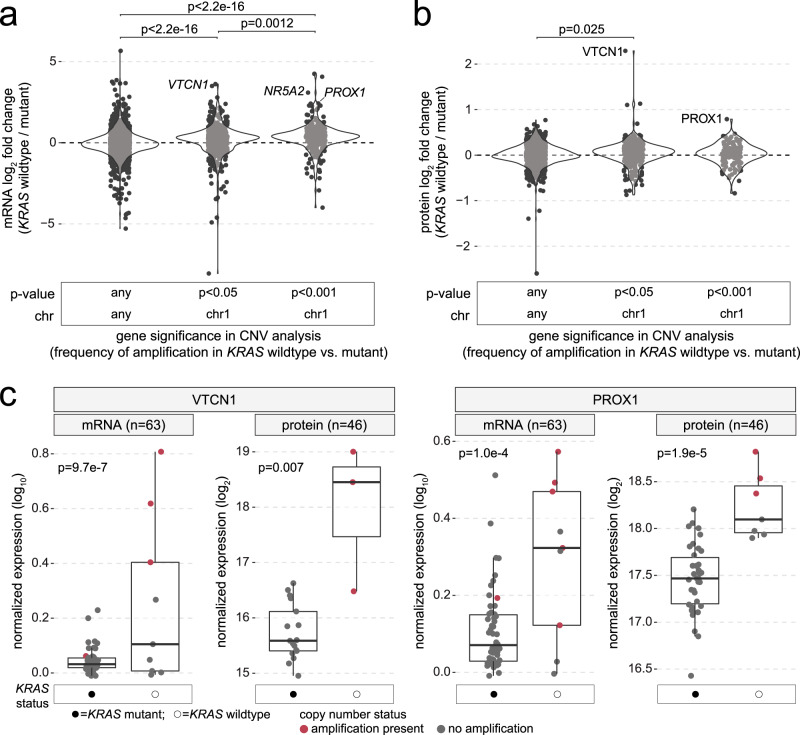
*VTCN1* and *PROX1* are increased in *KRAS* wildtype mPDAC at both mRNA and protein levels. **a** Violin plots showing distribution of mRNA expression fold changes (log_2_; *KRAS* wildtype vs. mutant) for genes grouped according to results of CNV analysis between *KRAS* wildtype versus mutant tumors. Left to right: genes located on any chromosome with no difference in rate of copy amplification in *KRAS* wildtype tumors, genes located on chr1 with a significantly higher rate of copy amplification in *KRAS* wildtype tumors above thresholds of *p* < 0.05 and *p* < 0.001. Each dot represents a gene. Two-tailed Wilcoxon mean rank-sum *p* values are shown. **b** Violin plots showing the distribution of protein-level fold changes (log_2_; *KRAS* wildtype vs. mutant) for genes grouped according to results of CNV analysis between *KRAS* wildtype versus mutant tumors. Two-tailed Wilcoxon mean rank-sum *p* values are shown. **c** Box plots comparing mRNA and protein levels between *KRAS* wildtype and mutant groups for *VTCN1* (mRNA: *KRAS* wildtype *n* = 9, *KRAS* mutant *n* = 54; protein: *n* = 3 and *n* = 17) and *PROX1* (mRNA: *n* = 9, *n* = 54; protein: *n* = 7 and *n* = 38). Each dot represents a sample, and dots are colored based on whether copy amplification of the gene was present. Box plots indicate median (central line), 25–75% IQR (bounds of box), and whiskers extend from box bounds to the largest value no further than 1.5 times the IQR. Two-tailed Wilcoxon mean rank-sum *p* values are shown. Source data are provided as a Source Data file.

We next sought to determine the degree by which *KRAS* wildtype-specific copy number amplification of chr1 correlated with differential mRNA expression patterns. For genes included in the exploratory analysis of copy number amplifications in *KRAS* wildtype tumors (*n* = 2987), significantly higher mRNA L2FC values were observed for genes located on chr1 that had significant (*p* < 0.05) rates of amplification in *KRAS* wildtype tumors (median L2FC = 0.27, *p* < 2.2e–16), and increases in L2FC were even more pronounced when using a more stringent CNV analysis *p* value cut-off of *p* < 0.001 (genes with highest differences in copy gain frequency between *KRAS* wildtype vs. mutant tumors; median L2FC = 0.38, *p* < 2.2e–16; Fig. 4a). Among genes with highest mRNA L2FC values and increased amplification frequency in *KRAS* wildtype tumors were *NR5A2* (chr1q; L2FC = 3.1, DEA *p* = 6.4e–8, CNV *p* = 9.0e–4), *PROX1* (chr1q; L2FC = 2.4, DEA *p* = 1.0e–4, CNV *p* = 9.0e–4) and *VTCN1* (chr1q; L2FC = 3.5, DEA *p* = 9.7e–7, CNV *p* = 0.023), a cell surface receptor that functions to inhibit T-cell activation. To further explore the effect of chr1q amplifications in *KRAS* wildtype PDAC, we performed mass spectrometry (MS)-based proteome profiling of PanGen patient tumor samples. Across all 7293 genes assayed in the MS dataset, protein and mRNA levels were significantly correlated (rho = 0.28, *p* < 2.2e–16). We calculated protein L2FC values for genes included in the analysis of copy number amplification in *KRAS* wildtype tumors that were available in the MS dataset (*n* = 1298; Supplementary Data 6). In agreement with the mRNA data, genes located on chr1 that had significant (*p* < 0.05) frequency of amplification in *KRAS* wildtype tumors showed higher L2FC (*KRAS* wildtype / mutant) values (median L2FC = 0.05, *p* = 0.025), though this observation was not significant for genes on chr1 with highest (*p* < 0.001) frequency of amplification in *KRAS* wildtype tumors (median L2FC = 0.032, *p* = 0.50; Fig. 4b). While NR5A2 protein levels were not available in the MS dataset, we observed significantly higher protein levels in *KRAS* wildtype versus mutant tumors for VTCN1 (protein L2FC = 2.3, *p* = 0.007) and PROX1 (protein L2FC = 0.79, *p* = 1.9e–5; Supplementary Data 7 and Fig. 4c). In the validation PDAC cohorts, we observed significantly higher *PROX1* mRNA levels in *KRAS* wildtype tumors belonging to the COMPASS cohort (*p* = 0.012), though differences in *PROX1* mRNA levels were not observed in the Hartwig cohort (*p* = 0.39), nor for *VTCN1* in either COMPASS (*p* = 0.17) or Hartwig (*p* = 0.42; Supplementary Fig. 8). Thus, while results of the PanGen study demonstrate *KRAS* wildtype-specific amplification of chr1q that is linked to subsequent increases in both mRNA and protein levels, chr1q amplification may not be generalizable to all cohorts of *KRAS* wildtype mPDAC.

### Direct comparison between cholangiocarcinoma and *KRAS* wildtype mPDAC

**Fig. 5:**
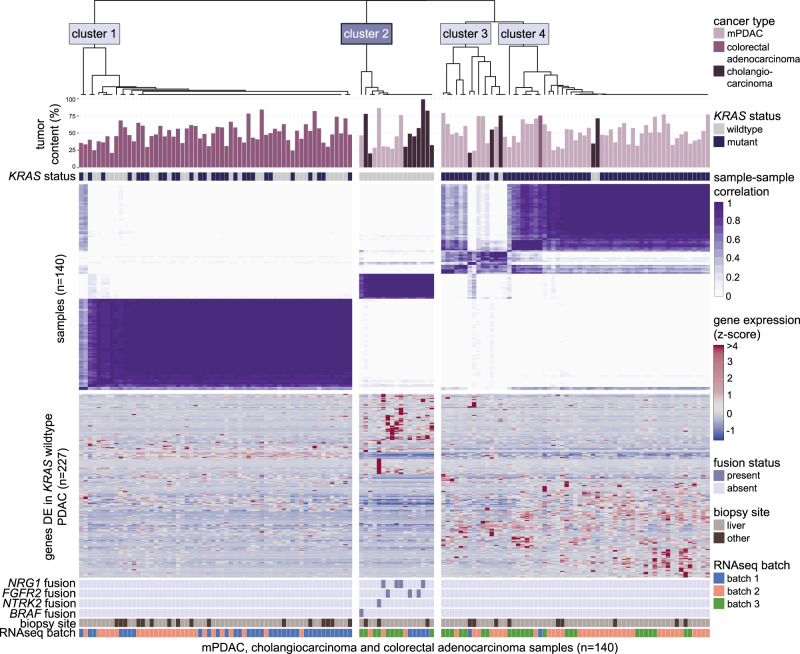
*KRAS* wildtype mPDAC samples show unique mutation and expression patterns that are shared with cholangiocarcinoma samples. Upper heatmap (purple/white) shows results of consensus clustering of mPDAC (*n* = 63), metastatic cholangiocarcinoma (*n* = 14), and metastatic colorectal adenocarcinoma (*n* = 63) samples based on mRNA expression levels (z-score) of the *KRAS* mutation status signature genes (genes found to be differentially expressed in *KRAS* wildtype mPDAC samples; *n* = 227). Upper bars indicate tumor content levels for each sample, with upper grid showing *KRAS* mutation status. Lower heatmap (blue/red) shows expression patterns of the genes used for clustering. Bottom grids show gene fusion events, biopsy sites, and RNAseq batches for each sample. Cholangiocarcinoma and *KRAS* wildtype mPDAC uniquely group together as part of Cluster 2.

While rare in mPDAC, oncogenic fusions involving genes such as *NRG1* and *FGFR2* are more frequently observed in cholangiocarcinoma^26,27^. The presence of such fusion events in *KRAS* wildtype mPDAC together with increased expression of genes expressed in cholangiocytes, while hypothesis-generating and not conclusive on their own, prompted us to directly investigate the relationship between *KRAS* wildtype mPDAC and cholangiocarcinoma samples, the latter of which received genome and transcriptome sequencing as part of the POG trial (*n* = 14; NCT02155621). We also included metastatic colorectal adenocarcinoma samples from the POG trial (*n* = 63) as an additional comparator, as colorectal adenocarcinoma represented an additional *KRAS* mutation-containing carcinoma cohort that were frequently biopsied from liver metastases (75% of samples biopsied from the liver, compared to 79% of cholangiocarcinoma and 86% of mPDAC samples). Consensus clustering of all PDAC, cholangiocarcinoma, and colorectal adenocarcinoma samples based on the expression of the *KRAS* mutation status signature genes (genes conservatively DE in *KRAS* wildtype vs. mutant mPDAC; *n* = 227) revealed an optimal solution based on four clusters (Fig. 5 and Supplementary Fig. 9). The majority of colorectal adenocarcinoma samples (98%; 62/63) clustered independently of mPDAC and cholangiocarcinoma samples and encompassed all samples in Cluster 1. Cluster 2 contained 89% (eight of nine; *p* = 4.6e–10) of *KRAS* wildtype mPDACs as well as the highest proportion of cholangiocarcinoma samples (64%; nine of 14; *p* = 1.4e–8) compared to all other clusters, further supporting the high transcriptional similarity between *KRAS* wildtype mPDAC and cholangiocarcinoma. Moreover, among the oncogenic fusions identified in mPDAC samples, we observed fusions involving *FGFR2* (two patients; fusion partners *BICC1* and *SORBS1*) and *NRG1*-*ATP1B1* (one patient) in three of 14 (21%) cholangiocarcinoma samples, all of which were contained in Cluster 2. No such oncogenic fusions were detected in colorectal adenocarcinoma samples. Clusters 3 and 4 contained mostly mPDAC samples (12/15 (80%) and 43/46 (93%), respectively) and differed in terms of the number of Moffitt basal-like samples contained in each cluster (zero of 15 (0%), and 15/15 (100%) basal-like mPDAC samples in Clusters 3 and 4, respectively). Tumor content (TC) values were similar between clusters (median TC 50%, 45%, 55 and 51% in Clusters 1, 2, 3, and 4, respectively; *p* = 0.50). While three separate RNA-sequencing (RNAseq) batches were encompassed by the three cancer types, pre-clustering batch correction was unable to be performed as the distribution of batches was skewed between cancer types (colorectal adenocarcinoma samples containing no “batch 3” samples and mPDAC samples containing no “batch 1” samples), which would result in dilution of true between-cancer type signal when batches are corrected for. Instead, we noted that no single batch dominated any of the four clusters, and Cluster 2 contained representation from all three batches, indicating that the RNAseq batches did not confound the clustering solution. Clustering based on cell composition landscape, estimated for each sample using xCell^28^, did not show meaningful differences nor similarities between cancer types, apart from the enrichment of one cell composition cluster (including stroma and fibroblast components) among low TC samples (*p* = 0.0045; Supplementary Fig. 10).

To further assess the genomic similarities between cholangiocarcinoma and *KRAS* wildtype mPDAC, we examined the spatial distribution of CNV events across chr1 and chr8 in the POG cholangiocarcinoma samples (Supplementary Fig. 11). Similar to *KRAS* wildtype mPDAC, amplification of chr1q was observed in approximately 20% of cholangiocarcinoma tumors (Supplementary Fig. 11a). Meanwhile, amplification of chr8q was not as frequent (approximately 14% of tumors) in cholangiocarcinoma (Supplementary Fig. 11b) compared to *KRAS* wildtype mPDAC.

To validate our finding of distinct mRNA-based similarities between cholangiocarcinoma and *KRAS* wildtype mPDAC, we leveraged RNAseq data for cholangiocarcinoma (*n* = 25) and mPDAC (*n* = 46) samples from the Hartwig validation dataset and performed an identical consensus clustering analysis. First, DEA of *KRAS* wildtype versus mutant mPDAC in the Hartwig dataset resulted in a list of 245 DE (absolute L2FC > 2.5, *p* = 0.005) genes, 190 (78%) of which were upregulated. Consensus clustering of these DE genes revealed an optimal solution based on four clusters (Supplementary Fig. 12). We again observed a distinct cluster (Cluster 1) containing the majority of *KRAS* wildtype mPDAC (six of eight (75%); *p* = 3.0e–6) and cholangiocarcinoma (15/25 (60%); *p* = 6.4e–4) samples. Oncogenic fusion events, which included *BRAF-FAM129A* (one patient with *KRAS* mutant disease) in mPDAC and *FGFR2* (fusion partners *WAC* (one patient), *PKD2L1* (one patient), *TENC1* (one patient), and *TBC1D4* (one patient)) in cholangiocarcinoma, were not uniquely contained in Cluster 1. TC values were similar across clusters (*p* = 0.11), while 31/46 (67%) and 16/24 (67%) of mPDAC and cholangiocarcinoma samples (respectively; with biopsy site missing for one cholangiocarcinoma sample) were biopsied from the liver. Taken together, these data provide evidence toward a striking molecular similarity between metastatic cholangiocarcinoma and *KRAS* wildtype mPDAC that is not shared with *KRAS* mutant mPDAC, while being observed in two orthogonal study cohorts of patient tumor samples.

## Discussion

Previous studies have established a marked distinction between *KRAS* wildtype and mutant mPDAC that involves an increased frequency of fusion events^5,29^ in patients with *KRAS* wildtype mPDAC. Results from the PanGen cohort of 63 patients with mPDAC support this observation, as somatic fusion events were identified in 67% of patients with *KRAS* wildtype tumors (with no known oncogenic fusions detected in patients with *KRAS* mutant tumors). While previous studies have identified increased survival among patients with *KRAS* wildtype PDAC^30^, the survival advantage associated with *KRAS* wildtype tumors in the PanGen cohort is likely biased by treatment differences between the two groups, as approximately half of the patients with *KRAS* wildtype tumors received therapy targeted at driver fusions. Given the discrepancies in prognostic significance of *KRAS* mutation status in the COMPASS and Hartwig validation mPDAC cohorts, it remains unclear how differences such as study design, patient demographics, treatments, or underlying molecular alterations may attribute to different survival patterns of patients with *KRAS* mutant mPDAC, and to what extent the small sample size of *KRAS* wildtype groups introduces unintended biases that may skew survival data. In any case, given the actionability and frequency of oncogenic fusion events among patients with *KRAS* wildtype tumors, incorporating panel-based *KRAS* mutation testing into the clinical management of patients newly diagnosed with PDAC, with subsequent NGS deployed for the ~10% of patients with *KRAS* wildtype PDAC, may prove beneficial for maximizing patient survival moving forward. From a health economics standpoint, in which the cost and logistics of NGS remain a barrier to delivering personalized care^31^, this reflexive strategy would aid in identifying the subset of patients who are most likely to benefit from molecular-targeted therapies.

Frequent copy number amplification of chr1q encompassing transcription factors *NR5A2* and *PROX1* in *KRAS* wildtype tumors provides important implications for the future development of therapeutic strategies targeting this subgroup of mPDAC. *NR5A2* has been established as a promising therapeutic target due to its known role in pancreatic cancer, including cell cycle regulation and early development^14^. The data presented here indicate a striking association between *KRAS* wildtype mPDAC and *NR5A2* in the PanGen study cohort, both in terms of copy number amplification and mRNA expression patterns. *PROX1* encodes a homeobox transcription factor with critical roles in cellular development, and has been linked to both oncogenic and tumor suppressive roles in different cancers^17^ while being a marker of better prognosis in gastric and pancreatic cancers^18,32^. Here, we show significant enrichment of *PROX1* copy number amplification in *KRAS* wildtype PDAC that is coupled with significant increases in both mRNA and protein levels of PROX1. From these data, we highlight the potential oncogenic driver role of *PROX1* in *KRAS* wildtype mPDAC as an exciting topic that warrants future in vivo research. Taken together, our results from the PanGen trial warrant further experimental investigation into the selective sensitivity of chr1q-amplified *KRAS* wildtype mPDAC cells to PROX1 and NR5A2 inhibition.

Basal-like and classical subtypes of PDAC each relate to a set of 25 genes identified in the original Moffitt subtyping manuscript^22^. Basal-like samples are characterized by relatively high and low expression of basal-like and classical genes, respectively, while classical samples show the opposite expression pattern (low basal-like, high classical expression) and discordant or “intermediate” samples show intermediate expression of both basal-like and classical genes^23^. Here, we discovered that some *KRAS* wildtype mPDAC do not conform to the expected Moffitt subtype expression patterns, as *KRAS* wildtype tumors received “classical” subtype calls using the published tool^33^ yet showed markedly low expression of classical genes in the PanGen cohort as well as the Hartwig validation dataset. As clinical PDAC research moves toward incorporating subtype-based information into first-line treatment decision-making^11,12^, it is worth considering the *KRAS* mutation status of patients, as our data indicate that some classical-subtype *KRAS* wildtype tumors may not conform to the expression-based phenotype observed in classical subtype *KRAS* mutant tumors.

Similarities between cholangiocarcinoma and PDAC have long been recognized and can present a diagnostic histopathology challenge. Both cancer types arise from epithelial cells of the pancreatobiliary system, share morphological and histological similarities, and in the case of mPDAC lesions of the liver, physiologically overlap. While studies have aimed at determining immunohistochemistry markers that distinguish PDAC from cholangiocarcinoma^34^, low sensitivity and specificity has impeded their adoption into routine pathology. Here, we demonstrate that similarities between these two cancer types on a molecular level are influenced by *KRAS* mutation status, as cholangiocarcinoma and *KRAS* wildtype mPDAC shared genomic (*NRG1*, *FGFR2* fusions and amplification of chr1q) and transcriptomic (upregulation of genes associated with cholangiocytes) features and clustered together independently of *KRAS* mutant mPDAC when clustered based on a set of *KRAS* mutation status signature genes. Moreover, using an independent cohort of metastatic cholangiocarcinoma and mPDAC from the Hartwig study, we validated our findings of the transcriptional similarity between cholangiocarcinoma and, specifically, *KRAS* wildtype mPDAC tumors. From a clinical perspective, immediate implications regarding the molecular similarity shared between *KRAS* wildtype mPDAC and cholangiocarcinoma are limited, as both diseases often receive platinum-based first-line treatment regimens (containing oxaliplatin and cisplatin, respectively). Instead, these findings may be relevant to ongoing and future clinical trials of cholangiocarcinoma treatment strategies. For instance, results of contemporary clinical trials investigating FGFR2 inhibition in cholangiocarcinoma, including infigratinib (NCT03773302) and futibatinib (NCT04093362), could perhaps be extrapolated to patients with *FGFR2* fusion positive, *KRAS* wildtype PDAC.

Our analysis of *KRAS* wildtype mPDAC bears several important limitations. From a clinical standpoint, it is difficult to draw a conclusion from a small cohort size such as the PanGen cohort of 63 patients, and our results are therefore hypothesis-generating. Rare occurrence coupled with challenges in core-needle biopsy and rapid disease progression hinder successful enrollment and sequencing of patient-derived mPDAC tumor samples, and the PanGen trial represents an ongoing clinical effort that encompasses multiple treatment centers and approximately 5 years of patient enrollment. Use of the COMPASS (*n* = 195) and Hartwig (*n* = 113) validation mPDAC cohorts was an important step to mitigating sample size limitations, and our finding of molecular similarities shared between cholangiocarcinoma and *KRAS* wildtype mPDAC was able to be validated in this way. Our study consisted solely of metastatic disease cohorts. High frequency of liver biopsy across the mPDAC, cholangiocarcinoma, and colorectal adenocarcinoma datasets reduced bias during the RNAseq clustering analysis. As resectable PDAC datasets such as TCGA, ICGC, and CPTAC are derived from pancreas biopsies, such datasets were not included and the applicability of our study results to early-stage PDAC remains an open question. Another limitation yet important observation of our study was the stark genomic differences between mPDAC cohorts, namely, fusion rates among *KRAS* wildtype samples (67% in PanGen, 31% in Hartwig and 6% in COMPASS) and fused genes (*NRG1* (three patients) and *NTRK2* (one patient) unique to PanGen; *NTRK3* (one patient) and *ALK* (one patient) unique to Hartwig). Furthermore, *KRAS* wildtype-specific chr1q amplification was only observed in the PanGen cohort. While such genomic events may not be generalizable across all cohorts of *KRAS* wildtype mPDAC, they highlight a diverse landscape of actionability and the importance of broad genomic testing for patients with *KRAS* wildtype mPDAC.

In conclusion, our results provide a comprehensive characterization of the genomic and gene expression landscape of *KRAS* wildtype tumors that furthers our understanding of the diverse underlying biology as well as subtyping-based profiles of this subgroup of mPDAC. The discovery of distinct amplification of chr1q, affecting mRNA and protein levels of transcription factor PROX1, provides hypotheses toward future therapeutic targeting of a subset of *KRAS* wildtype mPDAC. Furthermore, molecular concordances between *KRAS* wildtype mPDAC and cholangiocarcinoma provide impetus toward the future adoption of cholangiocarcinoma-specific treatment strategies in the clinical management of *KRAS* wildtype mPDAC. Overall, these data provide a significant rationale toward incorporating *KRAS* mutation status as part of standard-of-care testing in PDAC.

## Methods

### PanGen/POG study enrollment

PanGen (Prospectively Defining Metastatic Pancreatic Ductal Adenocarcinoma Subtypes by Comprehensive Genomic Analysis; NCT02869802) and POG (BC Cancer Personalized OncoGenomics; NCT02155621) trials are approved by the University of British Columbia Research Ethics Committee (REB# H14-00291 and H12-00137) and conducted in accordance with international ethical guidelines. Written informed consent was obtained from each patient prior to molecular profiling. For the discovery mPDAC cohort, all patients (*n* = 63) were enrolled and analyzed as part of the PanGen trial. Patient enrollment occurred between October 2016 and May 2021. All PanGen patients received tumor biopsy and sequencing prior to initiating treatment for their metastatic (unresectable) disease. All patients were confirmed as having a primary tumor located on the pancreas, with subsequent histopathological confirmation of PDAC. Metastatic (unresectable) tumors were biopsied for sequencing analysis.

Patients with metastatic colorectal adenocarcinoma (*n* = 63) and cholangiocarcinoma (*n* = 14) were enrolled as part of the POG trial. Cholangiocarcinoma samples were noted as either liver intrahepatic cholangiocarcinoma (*n* = 2), bile duct cholangiocarcinoma (*n* = 6), or liver cholangiocarcinoma (*n* = 6) by pathology review. For patients with metastatic colorectal cancer and cholangiocarcinoma, enrollment on the protocol could occur during any line of therapy if patients were deemed to have a reasonable life expectancy to benefit from genomic sequencing. PanGen and POG samples did not receive laser-capture microdissection prior to sequencing. All sequencing data were housed using a secure computing environment. The PanGen and POG clinical trials are not directly linked to a specific treatment, but rather aim to assess response to genomics-guided therapy, with treatments selected at the discretion of the treating oncologist.

### PanGen/POG genome and transcriptome sequencing

WGS was performed on tumor and matched normal (blood) samples with target depths of 80× and 40×, respectively. For collection of whole-genome and transcriptome data, sequencing was performed using one of: HiSeq2500, HiSeqX, and NextSeq500. Bases were called using the following software from Illumina: Illumina Off-line Basecaller v1.9.4, Illumina bcl2fastq v1.8.3, v1.8.4, and v2.17.1.14. WGS libraries had reads trimmed to 75 base pairs (bp) and were aligned (hg19; GRCh37-lite) using BWA-mem v0.7.6a^35^ with default parameters. WGS duplicate reads were marked using sambamba v0.5.5^36^ with default parameters. RNAseq was performed on tumor samples with a target depth of 200 million reads. RNAseq reads were trimmed to 75 bp and aligned (GRCh37-lite) using STAR v2.7.3^37^, with parameters: *-chimSegmentMin 20 -outSAMmultNmax 1 -outSAMstrandField intronMotif -outFilterIntronMotifs RemoveNoncanonical*. RNAseq duplicate reads were marked using PicardTools v2.17.3. Raw reads counts were assigned to Ensembl 75 genes using Subread v1.4.6^38^, normalized for library depth and gene size (RPKM), and log_10_-transformed.

### PanGen mPDAC MS proteomics

MS-based proteomics sequencing of clinical (PanGen) tumor samples from patients diagnosed with mPDAC (*n* = 46) was performed using the SP3-CTP pipeline. 400 µL of supernatant containing protein in RLT buffer from sequencing pipeline was heated at 95 °C for 15 min with mixing at 1200 rpm. 50 µL of 400 mM chloroacetamide was added to each sample at room temperature and incubated for 30 min in the dark. Further sample preparation was as described previously^39^. MS2-TMT data were collected on a Thermo Orbitrap Eclipse mass spectrometer coupled with low pH LC-MS. As the previous data were collected on a Thermo Orbitrap Fusion, differences in instrument parameters are as follows: for MS1 scans application mode was peptide, advanced peak determination was true, Xcalibur AcquireX was off. For MS2 scans, 9 minimum points across the peak were defined, normalized AGC target was 100%, enhanced resolution mode was off, relaxed restrictions when too few precursors are found was true, intensity threshold range was 5000–1 × 10, auto PTE windows were not enabled, collision energy mode was fixed, the normalized AGC target was 100%, maximum injection time was auto, and enhanced resolution mode was off. Thermo RAW files were converted to mzML by ThermoRawFileParser v1.3^40^. Spectra were searched using the MSFragger search engine v3.3^41^ in FragPipe computational platform v16.0 against the UniProt Human proteome (20,371 sequences, downloaded July 16, 2021) database appended to a list of common contaminants. Identification parameters in MSFragger were specified as trypsin digestion, maximum of two missed cleavages allowed, minimum peptide length of 6, precursor mass tolerance of 20 ppm, and a fragment mass tolerance of 20 ppm. MS and MS/MS mass calibration, MS/MS spectral deisotoping, and parameter optimization were enabled. Cysteine carbamidomethylation (+57.0215), lysine TMT labeling (+229.1629), and peptide N-terminal TMT labeling (+229.1629) were included as fixed modifications. Methionine oxidation (+15.9949) and serine TMT labeling (+229.1629) were included as variable modifications. Search output was processed by Philosopher workflow^42^ and Percolator^43^. Proteins were filtered to 1% protein-level false discovery rate (FDR) using the best peptide approach and picked FDR target-decoy strategy. Data from multiple TMT plexes were summarized using TMT-Integrator^44^ (max_pep_prob_thres = 0.9, min_pep_prob = 0.9, min_purity = 0.5, min_percent = 0.05 and median centering normalization).

### PanGen/POG somatic mutation calling

Somatic SNV/indels were called using paired tumor/normal WGS libraries using a combination of Strelka v2.9.10^45^ and Manta v1.5.0^46^ with default parameters and genome build GRCh37. Variants were annotated using SnpEff v4.3^47^ with parameters *-v GRCh37.75 -canon -no-downstream -no-upstream -noLog -noStats -no-intergenic*. CNV events and tumor ploidy were called using Facets v0.6.0^48^ with default parameters. Copy number amplification was defined as a segment having a total copy number greater than or equal to twice the tumor ploidy. Copy number segment calls were mapped to Refseq genes using bedtools v2.26.0. Expressed somatic fusions were identified based on RNAseq data using Arriba v1.2.0^19^ (default parameters), with a separate STAR alignment (recommended fusion calling parameters: *-outFilterMismatchNmax 3 -chimSegmentMin 10 -chimOutType WithinBAM SoftClip -chimJunctionOverhangMin 10 -chimScoreMin 1-chimScoreDropMax 30 -chimScoreJunctionNonGTAG 0 -chimScoreSeparation 1 -alignSJstitchMismatchNmax 5 -1 5 5 -chimSegmentReadGapMax 3*). Fusion events were filtered for in-frame events reported at a confidence level of “high” by Arriba.

### PanGen mPDAC tumor mutational burden

For TMB calculations, VCF files were first converted to MAF format using vcf2maf v1.6.18 with default parameters. Variants were filtered to exclude those located outside of exons (using consensus exon regions GRCh37.p13; GCF_000001405.25, downloaded June 19, 2020) and any common variants identified by ExAC^49^ (ExAC_nonTCGA.r0.3.1.sites.vep.vcf.gz). Variants were further filtered based on established guidelines^50,51^, including VAF ≥ 0.05, tumor depth ≥ 25, and alternate allele count ≥ 3. Only missense, nonsense, and in-frame/frameshift variants were included for TMB calculation. TMB was calculated using the sum of all filtered mutations in a sample, and 32.102474 Mb was used for the TMB denominator^50^.

### PanGen mPDAC exploratory SNV/indel and CNV analyses

Differences in the frequency of SNV/indels were compared between *KRAS* wildtype and mutant groups for genes mutated in more than three patients across the cohort (*n* = 119 genes). For CNVs, copy number amplification frequencies were compared for genes with copy gains in more than three patients across the cohort (*n* = 3818), and homozygous deletion frequencies were compared for genes with homozygous deletion in more than three patients across the cohort (*n* = 1394). For each gene, mutation frequency between groups was compared using Fisher’s exact test, and *p* values were subjected to Benjamini–Hochberg multiple test correction. When comparing the distribution of CNV frequencies across chromosomes 1 and 8, chromosomes were binned using non-overlapping 100 kb bins. Copy status was then mapped to each bin, for each sample, using bedtools v2.26.0. Chromosome ideograms (hg19) were constructed using the chromosome gram-staining pattern (bands) data downloaded from the UCSC genome browser (https://genome.ucsc.edu/).

### PanGen mPDAC differential expression and enrichment analysis

Raw mRNA levels of protein-coding genes that were expressed (non-zero read count) in at least half of the samples (*n* = 18,416) were used as input for DEA (*KRAS* mutant vs. wildtype) by DESeq2^52^. Samples encompassed two separate RNAseq library protocol batches (*n* = 32 and 31), which were accounted for in the experimental design matrix using the following formula: *~ batch* + *kras_status*. For downstream enrichment analysis, a conservative DE threshold of adjusted *p* value < 0.005 and absolute L2FC > 2.5 was used to limit the number of input genes. Gene set enrichment analysis was performed separately on up- and downregulated DE genes using hypergeometric tests, in which genes were assessed for overlap with each of 32,284 gene sets obtained from the molecular signatures database (MSigDB^20^; downloaded April 2021). Hypergeometric test *p* values were subjected to Benjamini–Hochberg multiple test correction. L2FC (*KRAS* mutant vs. wildtype) of protein levels were calculated using DEqMS^53^ with default parameters and the experimental design formula: *~ kras_status*.

### PanGen/POG mPDAC, colorectal, and cholangiocarcinoma clustering analysis

Log_10_-transformed, RPKM-normalized gene expression values were converted to z-scores prior to clustering, for genes found to be DE (absolute L2FC > 2.5, *p* < 0.005) in the PanGen mPDAC cohort (*n* = 227), and clustering was performed using this set of *KRAS* mutation status signature genes. Consensus clustering of PanGen mPDAC (*n* = 63), POG cholangiocarcinoma (*n* = 14) and POG colorectal adenocarcinoma (*n* = 63) samples was performed using R v3.6.3 package ConsensusClusterPlus, with parameters *reps* = *50, pItem* = *0.8, pFeature* = *1, clusterAlg* = *”hc”, distance* = *”pearson”, seed* = *123*, and *maxK* = *6*. The optimal clustering solution (k = 4) was chosen based on the area under the cumulative distribution function (CDF) curve. We noted that a clustering approach based on principal components analysis of batch-corrected RNAseq data did not yield a meaningful comparison between cancer types (Supplementary Fig. 13), and this was expected as some technical batches were wholly contained within a single cancer type cohort.

### COMPASS and Hartwig mPDAC validation datasets

Unresectable PDAC samples in the COMPASS validation cohort (*n* = 195) were derived from patients enrolled and sequenced as part of the Comprehensive Molecular Characterization of Advanced PDAC For Better Treatment Selection (COMPASS; NCT02750657) trial. RNAseq (*n* = 195) and WGS (*n* = 195) data for COMPASS patients was generated and processed as described previously^11^. COMPASS fusion events were called using Arriba v1.2.0, with the same parameters as were performed in the PanGen cohort. Survival data were available for all COMPASS patients (*n* = 195). Unresectable PDAC and metastatic cholangiocarcinoma samples in the Hartwig dataset were accessed through the Hartwig Medical Foundation database. Hartwig WGS data for PDAC (*n* = 113) and cholangiocarcinoma (*n* = 25) was accessed in the form of Purple and Linx tool outputs. For fusion calls (Linx), fusions were filtered based on those with *reported* = *True* and *likelihood* = *High* fields. Hartwig RNAseq data for mPDAC (*n* = 46) and cholangiocarcinoma (*n* = 25) samples were processed using the same pipeline as PanGen/POG samples. For each mPDAC validation dataset, normalized gene expression values (FPKM for COMPASS, RPKM for Hartwig) were log_10_-transformed prior to analysis. All somatic mutation data were based on human genome build GRCh37 (hg19). For both COMPASS and Hartwig mPDAC RNAseq data, log fold change values for mRNA expression were generated using DESeq2, with the experimental design formula: *~kras_status*. OS and censorship data were available for 84 Hartwig patients with PDAC.

### Hartwig mPDAC and cholangiocarcinoma clustering analysis

Log_10_-transformed, RPKM-normalized gene expression values were converted to z-scores prior to clustering of Hartwig mPDAC (*n* = 46) and cholangiocarcinoma (*n* = 25) samples, for genes identified as DE (absolute L2FC > 2.5, *p* < 0.005; *n* = 245) in *KRAS* wildtype tumors in the Hartwig mPDAC cohort. Consensus clustering of all samples was performed using R v3.6.3 package ConsensusClusterPlus, with parameters *reps* = *50, pItem* = *0.8, pFeature* = *1, clusterAlg* = *”hc”, distance* = *”pearson”, seed* = *123*, and *maxK* = *6*. The optimal clustering solution (*k* = 4) was chosen based on the area under the CDF curve.

### PDAC subtyping

Classification of samples into the Moffitt basal-like and classical groups was performed using the RNAseq version of the Moffitt PurIST algorithm^33^. PurIST scores (basal-like probability values) were used to stratify patients into basal-like (score > 0.75), classical (score < 0.25) and intermediate (score [0.25–0.75]) subtype groups^23^. Basal-like (*n* = 25) and classical (*n* = 25) genes from the original Moffitt subtyping manuscript^22^ were used when directly investigating the expression values of Moffitt subtyping genes. Collisson and Bailey subtypes were determined separately using semi-automatic clustering approaches, in which R v3.6.3 package ConsensusClusterPlus was performed on all samples based on normalized expression of Collisson and Bailey subtyping genes, with parameters *reps* = *50*, *pItem* = *0.8*, *pFeature* = *1, clusterAlg* = *”hc”, distance* = *”pearson”, seed* = *123*, and *maxK* = *3* (Collisson) or *4* (Bailey) to quantify relatedness between samples. These inter-sample relatedness values, as output by ConsensusClusterPlus, were then subjected to hierarchical clustering and dendrograms were manually cut according to underlying subtyping gene expression patterns to produce the expected subtypes^25^. Collisson classical (*n* = 22), exocrine-like (*n* = 20), and quasi-mesenchymal (*n* = 20) genes from the original Collisson subtyping paper^8^ were used when assigning Collisson subtypes or directly investigating the expression values of Collisson subtyping genes. ADEX (*n* = 240), immunogenic (*n* = 370), squamous (*n* = 1061) and progenitor (*n* = 268) genes from the original Bailey subtyping paper^3^ were used when assigning Bailey subtypes or directory comparing expression values of Bailey subtyping genes. Metabolic subtypes were determined based on the relative expression of glycolytic (*GAPDH, ALDOA, PKM, ENO1, TPI1, PGK1, GPI, PGAM1, PFKP, PFKFB3, ENO2, PPP2R5D, PFKM, PFKFB4*) and cholesterogenic (*FDPS, FDFT1, DHCR24, EBP, IDI1, MVD, HMGCS1, SQLE, NSDHL, DHCR7, HMGCR, LSS, SC5D, MVK, HSD1787*) genes, as previously described^25^.

### Statistical analysis

Fisher’s exact tests were used to compare SNV/indel/CNV frequency and subtype calls between *KRAS* wildtype and mutant sample groups. Log-rank tests were used to calculate *p* values in the Kaplan–Meier analysis. Multivariate survival analysis was performed using Cox proportional hazards regression model, with *p* values based on the Wald statistic and selection of covariates was performed using a Forward Selection approach (covariate *p* value cut-off = 0.1). Wilcoxon mean rank-sum tests were used for two-group comparison of continuous variables. All group comparison tests were two-tailed. Wilcoxon signed-rank tests were used when testing whether the distribution of a continuous variable was symmetric about zero. A one-way ANOVA was used to test for differences in TC between clustering groups. Gene set enrichment analysis was performed based on one-tailed hypergeometric tests. All *p* values were subjected to Benjamini–Hochberg multiple test correction when three or more tests were performed. All analyses were performed using R v3.6.3.

### Reporting summary

Further information on research design is available in the Nature Research Reporting Summary linked to this article.

## Supplementary information

Supplementary Information

Description of Additional Supplementary Files

Supplementary Data 1

Supplementary Data 2

Supplementary Data 3

Supplementary Data 4

Supplementary Data 5

Supplementary Data 6

Supplementary Data 7

Reporting Summary

## Data Availability

Genomic data generated within the PanGen/POG and COMPASS studies are actively submitted to the European Genome-phenome Archive (EGA) under accession numbers EGAS00001001159 and EGAS00001002543, respectively. Data uploaded to EGA as part of the POG/PanGen study, including raw RNA and whole-genome sequencing files, will be made available to interested researchers while respecting patient privacy, and can be accessed through the BC Cancer Data Access Committee (https://ega-archive.org/dacs/EGAC00000000011; email address: tdoadmin@phsa.ca), which provides responses within 3–5 business days. Upon establishment and signing of the data transfer agreement, EGA data release can be expected within 3 business days. Once access has been granted, the period during which the data can be downloaded is flexible according to the downloader’s needs. Data access through EGA is on a limited use and project-specific basis. These data are available under restricted access in accordance with the ethical data regulations followed by the POG and PanGen trials. Hartwig data were accessed through the Hartwig Medical Foundation database (https://www.hartwigmedicalfoundation.nl/data/databank/). The UniProt Human proteome is available from https://www.uniprot.org/proteomes/UP000005640. Processed VTCN1 and PROX1 protein-level data are included as Supplementary Data 7. Raw protein data are available in the Proteomics Identifications Database (PRIDE) under accession number PXD036632. The remaining data are available within the article, Supplementary information, or Source Data file. Source Data are provided with this paper.

## Source data

Source Data
