# Supplementary material for: Integrative analysis of KRAS wildtype metastatic pancreatic ductal adenocarcinoma reveals mutation and expression-based similarities to cholangiocarcinoma
Source: Nat Commun. 2022 Oct 8;13:5941. doi: 10.1038/s41467-022-33718-7 (PMC9547977; doi:10.1038/s41467-022-33718-7)
Supplement: Supplementary file 1 — Supplementary Information [file 41467_2022_33718_MOESM1_ESM.pdf]

	KRAS mutant (N=54)	*KRAS* wildtype (N=9)	p value
Age, median (IQR)	60.9 (56.7-67.4)	51.4 (48.8-55.2)	0.03
Gender			
Female	20 (37%)	3 (33%)	1.00
Male	34 (63%)	6 (67%)	
Race/Ethnicity			
African	1 (2%)	1 (11%)	
Asian	15 (28%)	3 (33%)	
First Nations	1 (2%)	1 (11%)	0.43
Hispanic	1 (2%)	0	
White	32 (59%)	4 (44%)	
Unknown	4 (7%)	0	
Previous pancreatitis			
Yes	2 (4%)	1 (11%)	0.38
No	52 (96%)	8 (89%)	
History of diabetes			
Yes	14 (26%)	2 (22%)	1.00
No	40 (74%)	7 (78%)	
Family history of malignancy			
Yes	48 (89%)	9 (100%)	1.00
No	6 (11%)	0	
Location of primary tumor			
Head	21 (39%)	3 (33%)	
Neck	2 (4%)	2 (22%)	
Body	12 (22%)	2 (22%)	0.39
Tail	13 (24%)	1 (11%)	
Overlapping	4 (7%)	1 (11%)	
Not specified	2 (4%)	0	
Grade			
Grade 1	3 (6%)	0	
Grade 2	20 (37%)	4 (44%)	0.31
Grade 3	7 (13%)	2 (22%)	
Unknown	24 (44%)	3 (33%)	
Location of metastases			
Liver	46 (85%)	8 (89%)	
Lung	17 (31%)	1 (13%)	
Lymph nodes	25 (46%)	3 (33%)	-
Omentum	8 (15%)	1 (11%)	
Bone	5 (9%)	0	
Other	5 (9%)	1 (11%)	
Median CA 19-9 at baseline (U/mL; IQR)	4900 (90-29,000)	58 (18-495)	0.03
First-line chemo			
FOLFIRINOX	27 (50%)	5 (56%)	
FOLFIRINOX/irbesartan	1 (2%)	0	
Gemcitabine and nab-paclitaxel	14 (26%)	1 (11%)	0.24
Clinical trial* – intervention	10 (19%)	1 (11%)	
Clinical trial* – standard	2 (4%)	1 (11%)	
Afatinib**	0	1 (11%)	

	KRAS mutant (N=54)	*KRAS* wildtype (N=9)	p value
Median duration of first-line chemotherapy (IQR)[1]	4.30 (2.1-7.3)	9.53 (2.0-12.1)	0.18
Second-line chemo[2]			0.10
FOLFIRINOX	1 (2%)	0	
FOLFIRI/CAPIRI	4 (7%)	0	
FOLFOX/CAPOX	3 (6%)	0	
Capecitabine	2 (4%)	0	
Gemcitabine and nab-paclitaxel	5 (9%)	1 (13%)	
Gemcitabine and cisplatin	3 (6%)	1 (13%)	
Gemcitabine	3 (6%)	0	
Gemcitabine and SLC-0111***	2 (4%)	0	
Palbociclib	1 (2%)	0	
Afatinib	0	3 (33%)	
Erlotinib	0	1 (13%)	
Median duration of second-line chemotherapy (IQR)[2]	2.98 (1.33-5.27)	3.90 (0.97-8.47)	0.56
Third-line chemo			0.04
Capecitabine and nal-IRI/FOLFIRI	1 (2%)	1 (13%)	
Gemcitabine and nab-paclitaxel	0	1 (13%)	
Median duration of third-line chemotherapy (IQR)	1.03	1.07 (0.7-1.4)	1.00

Supplementary Table 1: Clinical characteristics of patients with *KRAS* wildtype and mutant mPDAC tumors. *A phase II randomized clinical trial where intervention arm involved gemcitabine, nab-paclitaxel, durvalumab, and tremelimumab, compared to control arm of gemcitabine and nab-paclitaxel (NCT02879318). **This patient declined cytotoxic chemotherapy and instead received targeted therapy. ***Phase Ib clinical trial of SLC-0111 with gemcitabine (NCT03450018). [1]Six patients in the *KRAS*-mutant group and three in the *KRAS*-wildtype group are continuing to receive first-line systemic therapy. [2]Two patients in the *KRAS*-wildtype group are continuing to receive second-line systemic therapy. Two-tailed Fisher's exact test p values are shown.

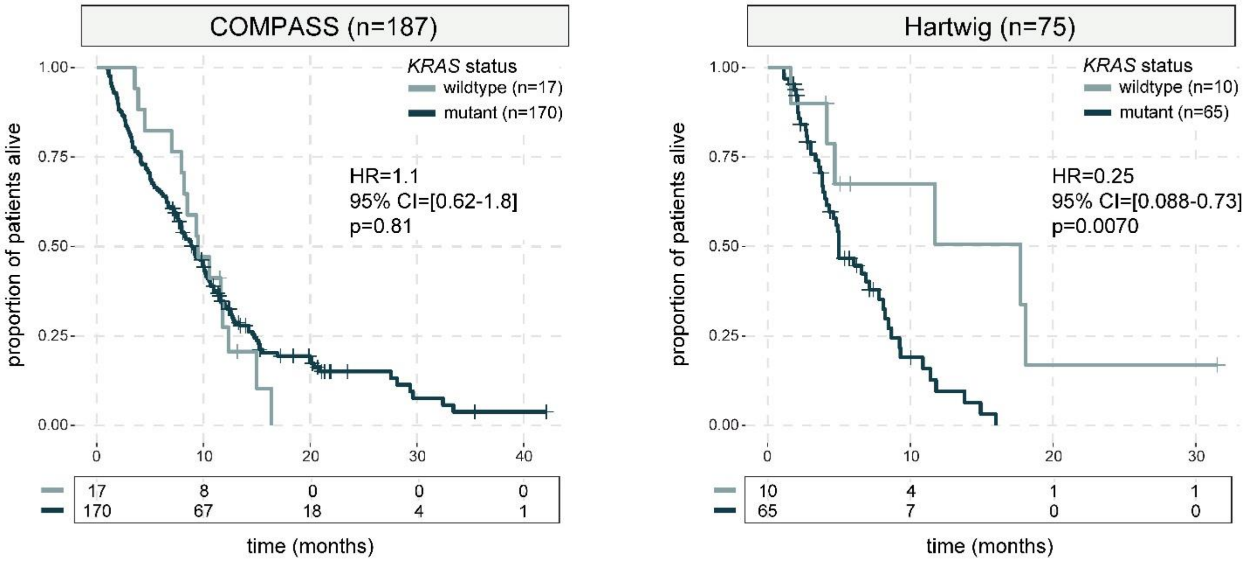

Supplementary Figure 1: Stratification of patients based on *KRAS* mutation status in the validation PDAC datasets. Kaplan-Meier curves comparing overall survival between *KRAS* mutant and wildtype samples in the COMPASS (left) and Hartwig cohorts of unresectable PDAC. Hazard ratio (HR), 95% confidence interval (CI) and log-rank p values are shown.

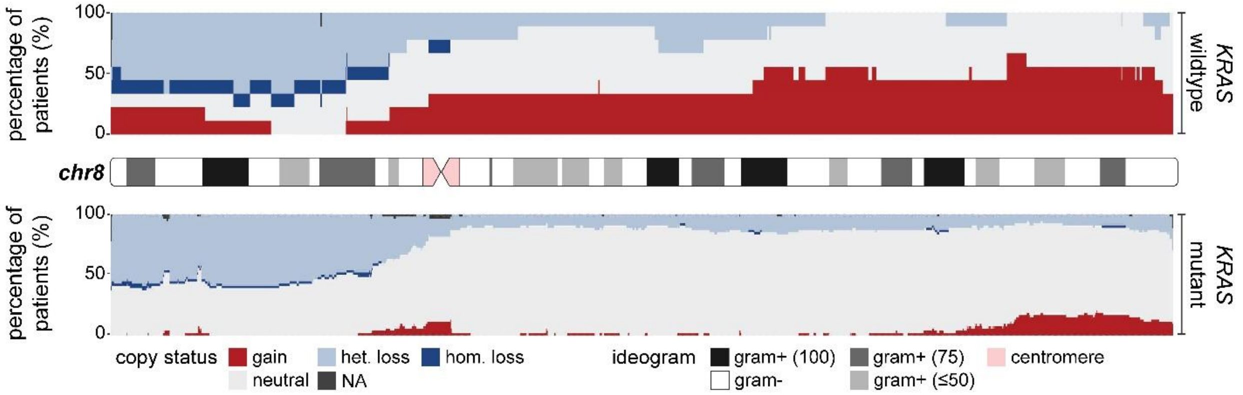

Supplementary Figure 2: Distribution of copy status along chromosome 8 in *KRAS* wildtype and mutant groups. Stacked bar plot indicating the distribution of copy number alterations along chromosome 8 (100kb bins) in *KRAS* wildtype (top) and mutant (bottom) groups in the PanGen cohort of unresectable PDAC (n=63). Center ideogram shows chromosome 8 staining and centromere location.

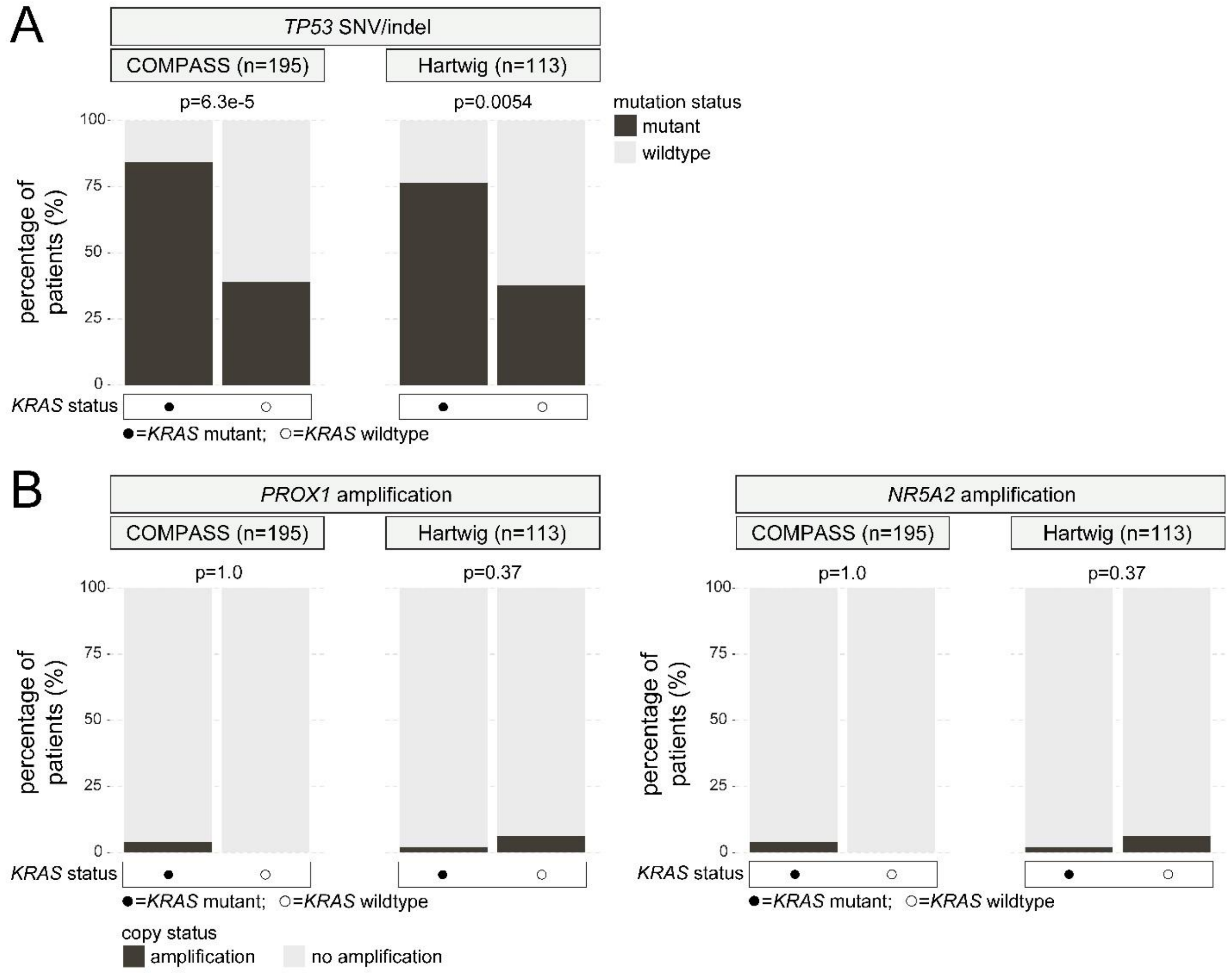

Supplementary Figure 3: Frequency of SNV/indels in *TP53* and copy number amplification of *PROX1* and *NR5A2* between *KRAS* wildtype and mutant groups in the validation PDAC cohorts. (A) Stacked bar plots comparing frequency of SNV/indels in *TP53* between *KRAS* wildtype and mutant groups in the COMPASS and Hartwig PDAC cohorts. Two-tailed Fisher's exact test p values are shown. (B) Stacked bar plots comparing frequency of copy number amplification in *PROX1* (left) and *NR5A2* (right) between *KRAS* wildtype and mutant groups in the COMPASS and Hartwig PDAC cohorts. Two-tailed Fisher's exact test p values are shown.

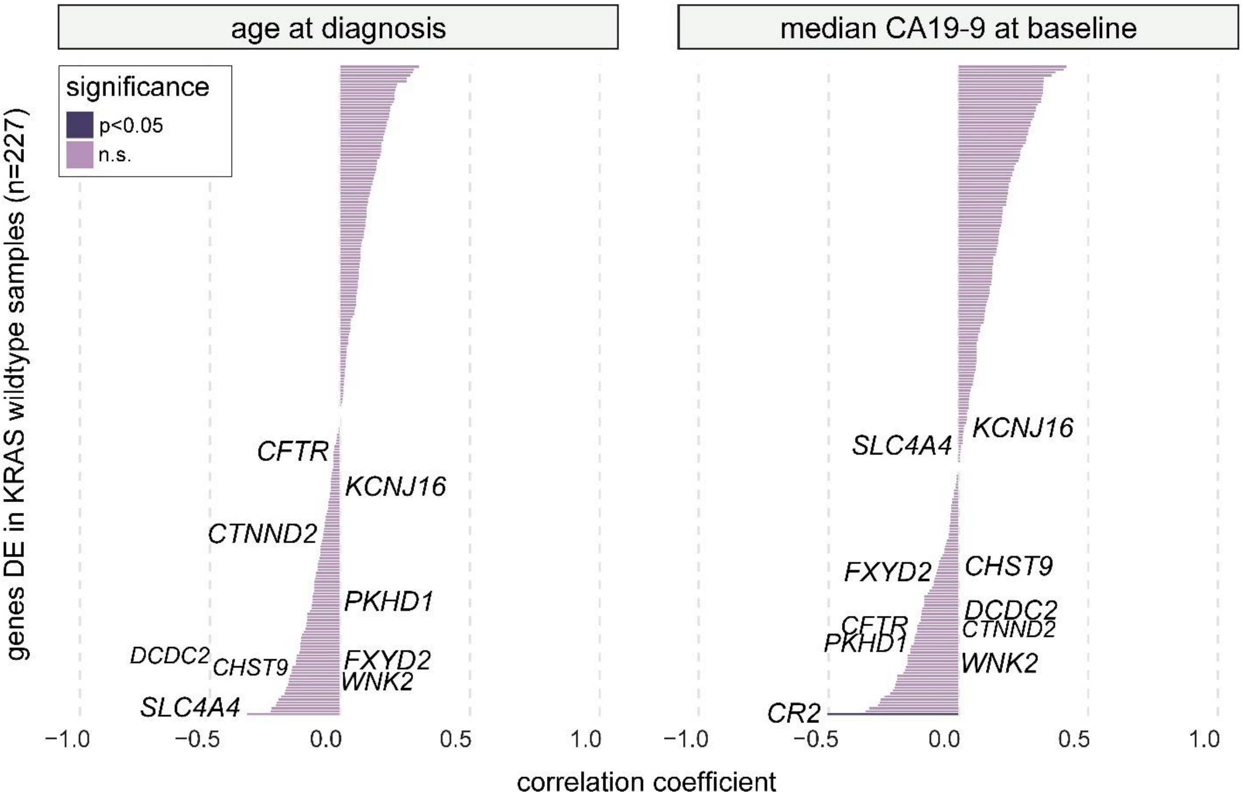

Supplementary Figure 4: Correlation between *KRAS* wildtype versus mutant DE genes and clinical variables associated with *KRAS* mutation status. Bar plots depicting two-tailed Spearman correlation coefficient values for each *KRAS* wildtype versus mutant DE gene (n=227) when compared versus age at diagnosis (left) and median CA19-9 at baseline (right). Genes that were DE and overlapped with the Aizarani *et al.* cholangiocyte gene set are labelled, along with *CR2*. *CR2* was the only gene that showed significant (p<0.05) correlation with either clinical variable. P values are two-tailed, and were subjected to Benjamini-Hochberg multiple test correction.

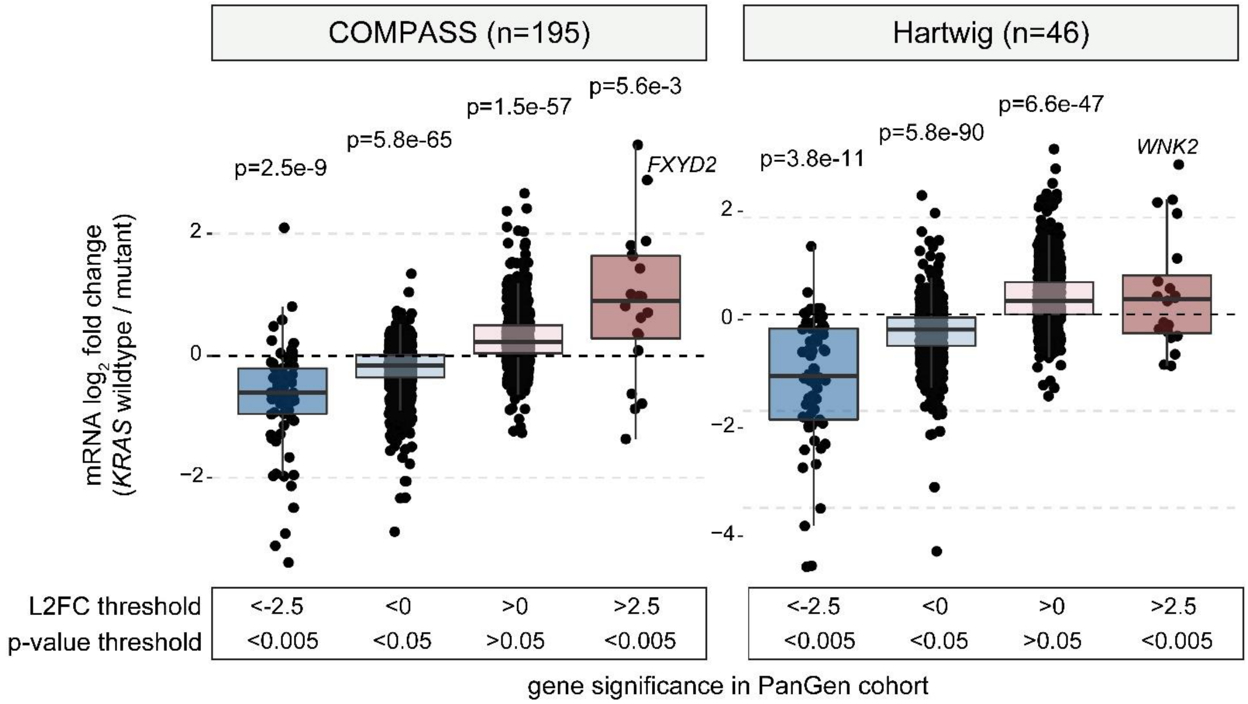

Supplementary Figure 5: Analysis of DE genes in the validation PDAC cohorts. Box plots showing distribution of log_2 fold change (L2FC) values (*KRAS* wildtype / mutant) for mRNA expression in the COMPASS (left) and Hartwig (right) validation cohorts, for genes that were significantly ($p<0.05$ or $p<0.005$) up (L2FC > 0 or 2.5) or down (L2FC < 0 or -2.5) regulated in *KRAS* wildtype samples in the PanGen cohort. Box plots indicate median (central line), 25-75% IQR (bounds of box) and whiskers extend from box bounds to the largest value no further than 1.5 times the IQR. Two-tailed Wilcoxon signed-rank test p values shown.

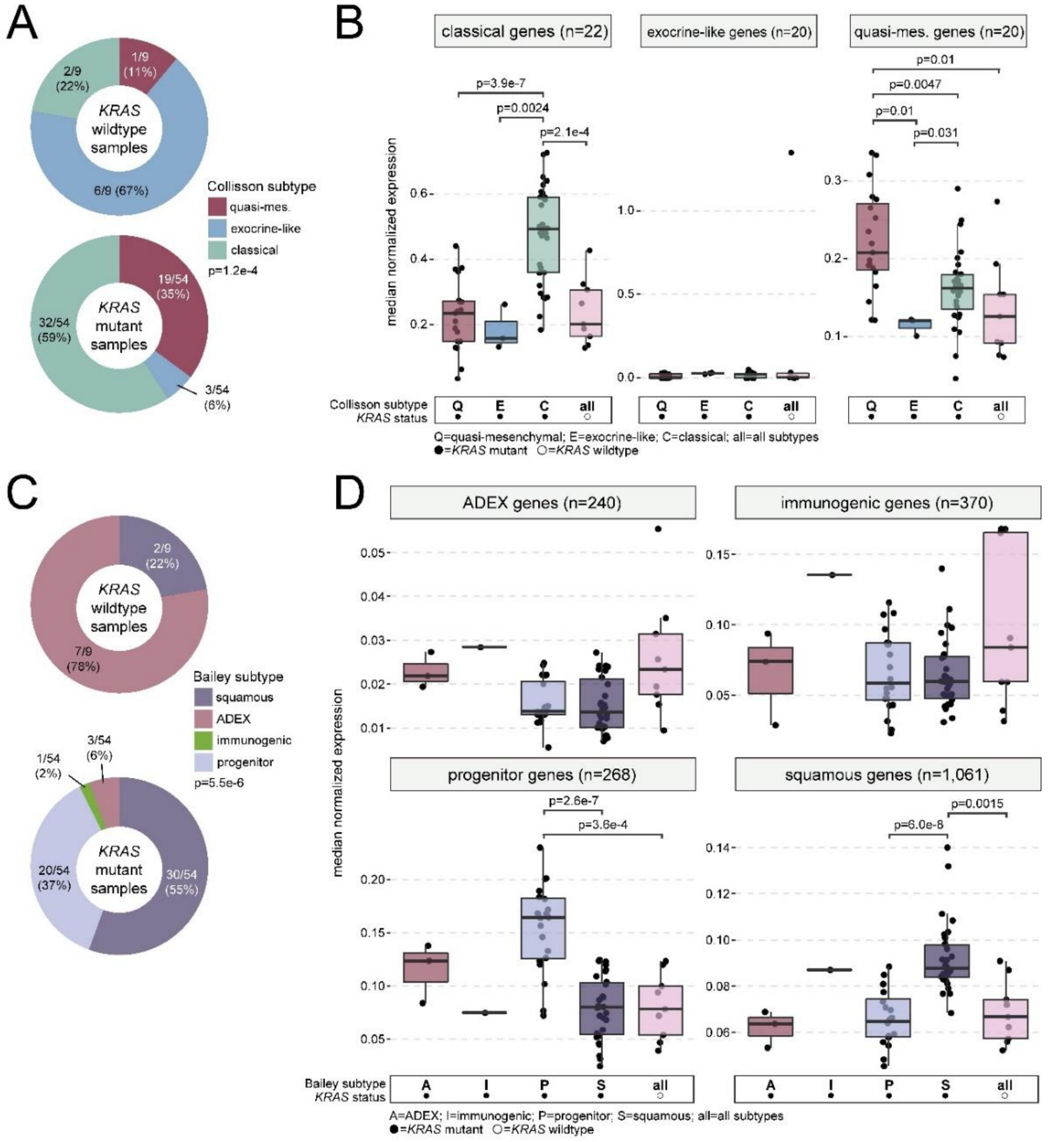

Supplementary Figure 6: Collisson and Bailey subtype and gene expression patterns between *KRAS* mutant and wildtype tumors in the PanGen PDAC cohort. (A) Pie charts showing distribution of Collisson subtypes between *KRAS* mutant and wildtype samples in the PanGen PDAC cohort. Two-tailed Fisher's exact test p value is shown. (B) Box plots comparing median mRNA expression levels of classical, exocrine-like and quasi-mesenchymal gene sets. Samples are grouped based on *KRAS* mutation status, while *KRAS* mutant samples are further stratified by their Collisson subtype calls. Left to right: *KRAS* mutant quasi-mesenchymal (n=19), *KRAS* mutant exocrine (n=3), *KRAS* mutant classical (n=32), *KRAS* wildtype (n=9). Box plots indicate median (central line), 25-75% IQR (bounds of box) and whiskers extend from box bounds to the largest value no further than 1.5 times the IQR. Two-tailed Wilcoxon mean rank-sum p values are shown. (C) Pie charts showing distribution of Bailey subtypes between *KRAS* mutant and wildtype samples in the PanGen PDAC cohort. Two-tailed Fisher's exact test p value is shown. (D) Box plots comparing median mRNA expression levels of ADEX, immunogenic, progenitor and squamous gene sets. Samples are grouped based on *KRAS* mutation status, while *KRAS* mutant samples are further stratified by their Bailey subtype calls. Left to right: *KRAS* mutant ADEX (n=3), *KRAS* mutant immunogenic (n=1), *KRAS* mutant progenitor (n=20), *KRAS* mutant squamous (n=30), *KRAS* wildtype

(n=9). Box plots indicate median (central line), 25-75% IQR (bounds of box) and whiskers extend from box bounds to the largest value no further than 1.5 times the IQR. Two-tailed Wilcoxon mean rank-sum p values are shown.

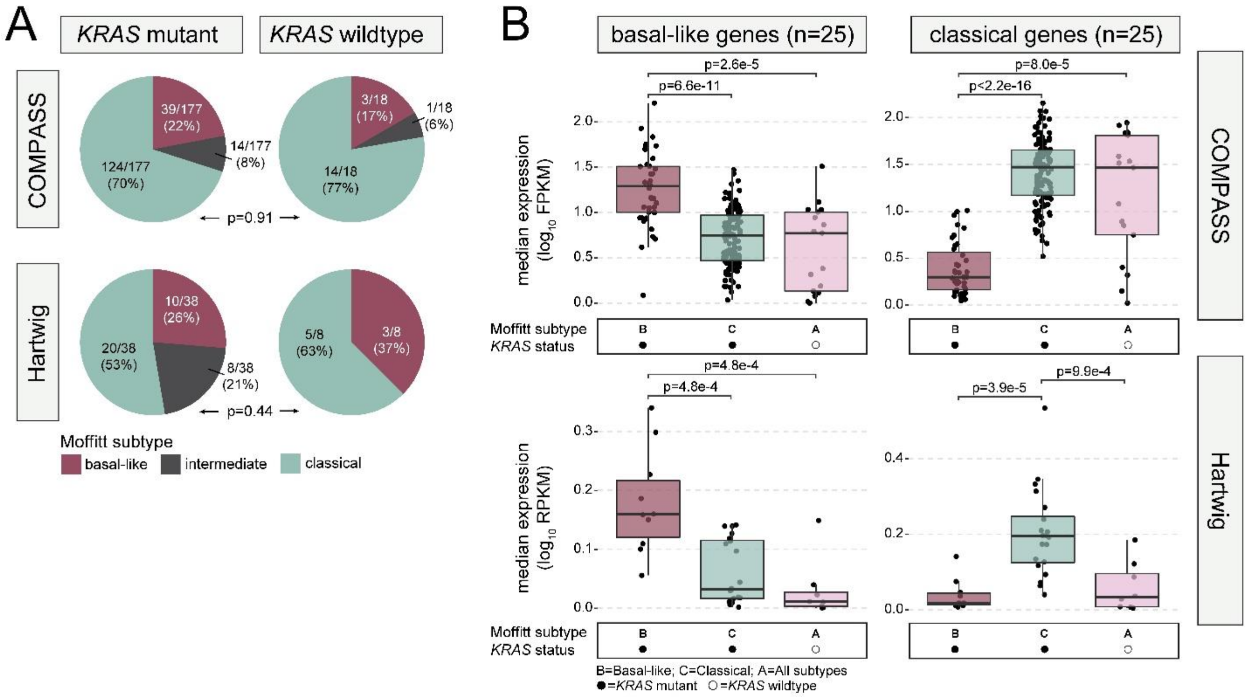

Supplementary Figure 7: Analysis of Moffitt subtype patterns between *KRAS* mutant and wildtype groups in the validation PDAC cohorts. (A) Donut plots showing the distribution of Moffitt subtype calls across *KRAS* wildtype (right) and mutant (left) groups in the COMPASS and Hartwig PDAC validation cohorts. Two-tailed Fisher's exact test p values are shown. (B) Box plots comparing median mRNA expression levels of Moffitt basal-like (left) and classical (right) genes across *KRAS* wildtype and mutant groups, with *KRAS* mutant groups stratified by Moffitt subtype, in each validation cohort. COMPASS: *KRAS* mutant basal-like (n=39), *KRAS* mutant classical (n=124), *KRAS* wildtype (n=18). Hartwig: *KRAS* mutant basal-like (n=10), *KRAS* mutant classical (n=20), *KRAS* wildtype (n=8). Box plots indicate median (central line), 25-75% IQR (bounds of box) and whiskers extend from box bounds to the largest value no further than 1.5 times the IQR. Two-tailed Wilcoxon mean rank-sum p values shown.

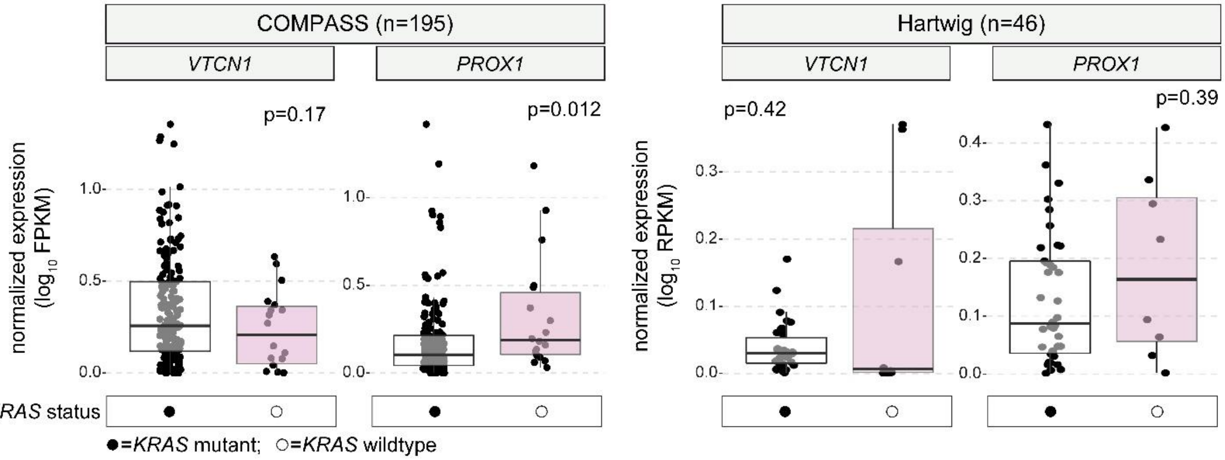

Supplementary Figure 8: Comparison of *VTCN1* and *PROX1* mRNA expression patterns between *KRAS* mutant and wildtype groups in the validation PDAC cohorts. Box plots comparing mRNA expression levels of *VTCN1* and *PROX1* between *KRAS* wildtype and mutant groups in the COMPASS (left) and Hartwig (right) validation cohorts. Box plots indicate median (central line), 25-75% IQR (bounds of box) and whiskers extend from box bounds to the largest value no further than 1.5 times the IQR. Two-tailed Wilcoxon mean rank-sum p values shown.

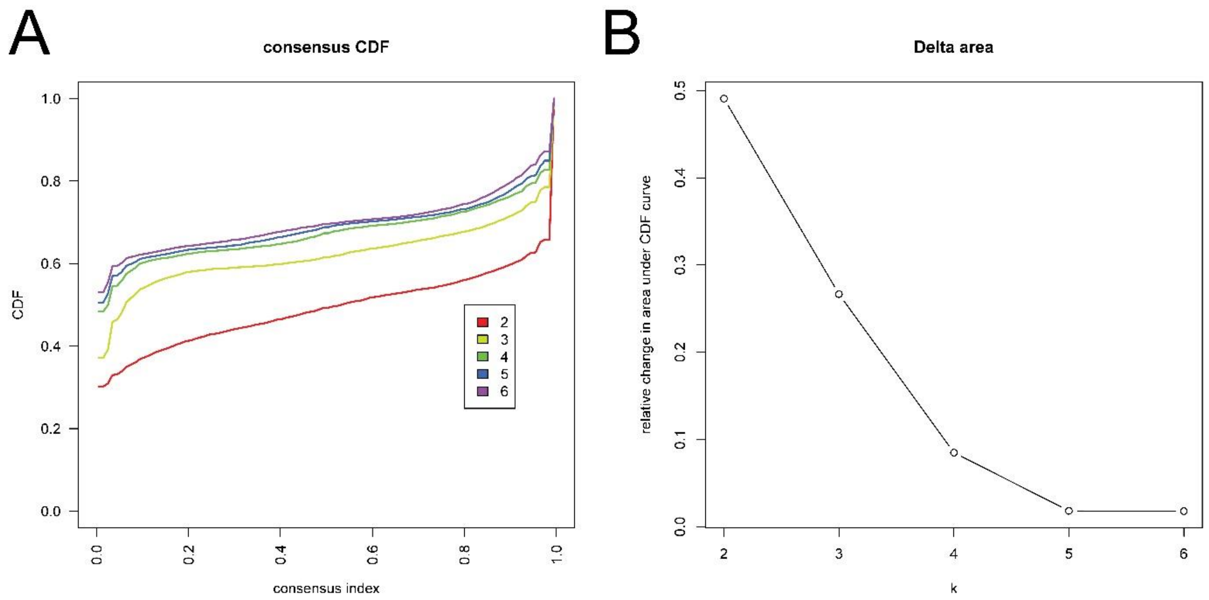

Supplementary Figure 9: Results of mRNA expression-based consensus clustering of metastatic PDAC (PanGen), cholangiocarcinoma (POG) and colorectal (POG) samples. (A) Cumulative distribution function (CDF) plot showing CDFs of the consensus matrix for each value of k (2-6, indicated by colors). Area under the CDF curve is indicative of clustering confidence. (B) Delta area plot showing the change in area under the CDF curve between values of k and their previous value (k-1).

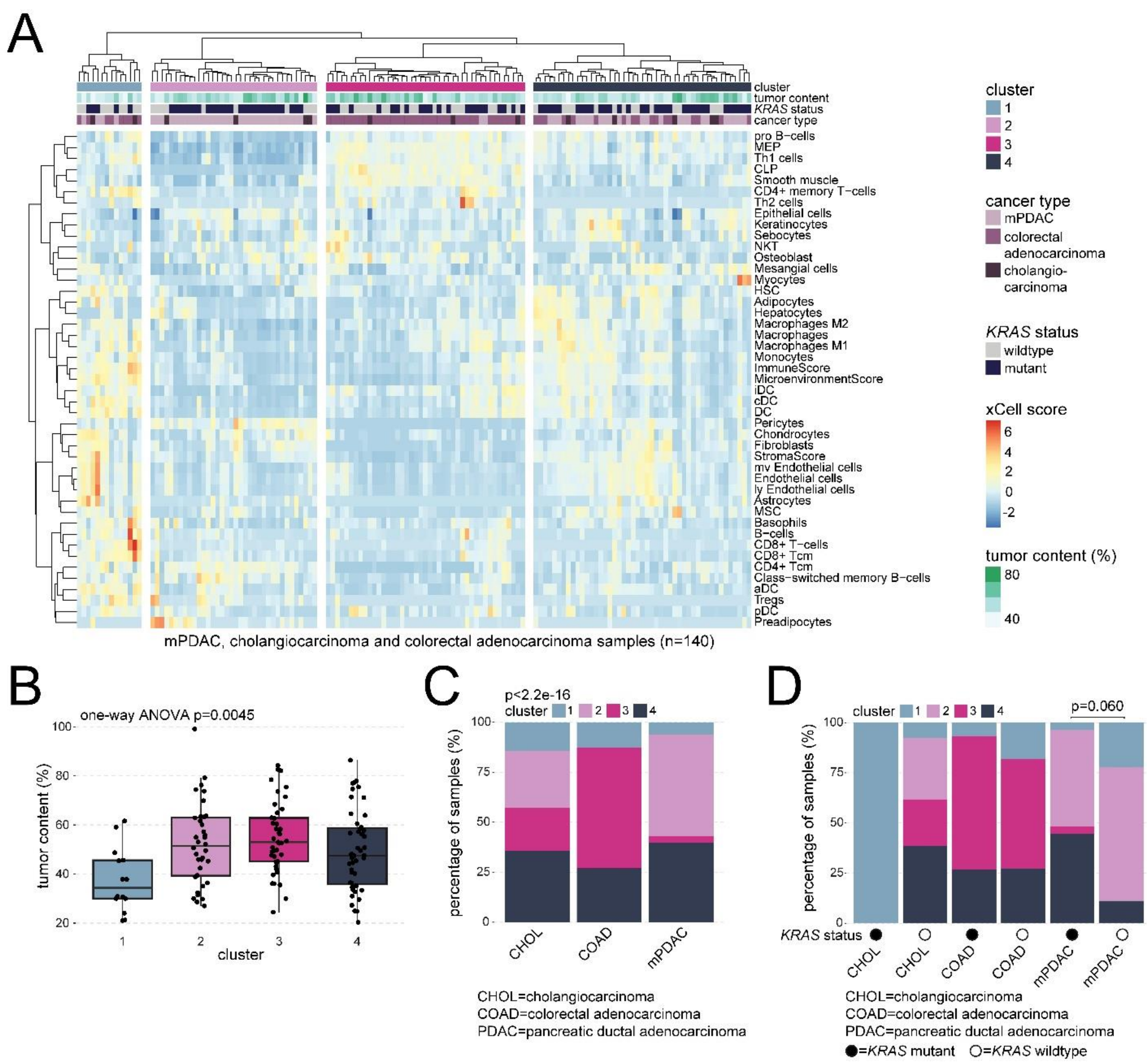

Supplementary Figure 10: Tissue cellularity landscape across tumor samples using xCell. (A) Heatmap showing cell type scores (z-score) across the mPDAC (n=63), cholangiocarcinoma (n=14) and colorectal adenocarcinoma (n=63) samples. Upper tracks indicate cluster membership, tumor content, *KRAS* mutation status and cancer type. (B) Boxplots comparing tumor content distribution between Clusters 1 (n=14), 2 (n=36), 3 (n=43) and 4 (n=47). Box plots indicate median (central line), 25-75% IQR (bounds of box) and whiskers extend from box bounds to the largest value no further than 1.5 times the IQR, and two-tailed one-way ANOVA p value is shown. (C) Bar plot indicating distribution of cluster membership across cholangiocarcinoma (CHOL; n=14), colorectal adenocarcinoma (COAD; n=63) and mPDAC (n=63) cancer types. Two-tailed Fisher's exact test p value is shown. (D) Bar plot showing distribution of cluster membership across cancer types, stratified by *KRAS* mutation status. Two-tailed Fisher's exact test p value is shown.

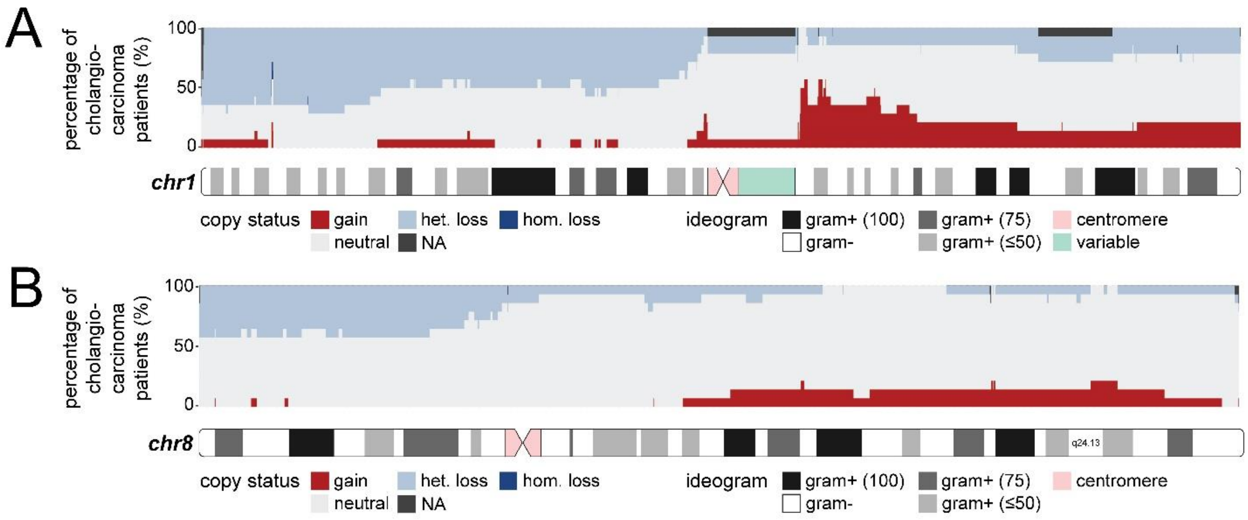

Supplementary Figure 11: Distribution of copy number status across chr1 and chr8 in POG cholangiocarcinoma samples. (A) Stacked bar plot showing CNV frequency across the length of chr1 in all POG cholangiocarcinoma samples (n=14). Bottom track shows the corresponding ideogram for chr1, with coloring based on reported gram-staining patterns. (B) Stacked bar plot showing CNV frequency across the length of chr8 in all POG cholangiocarcinoma samples (n=14). Bottom track shows the corresponding ideogram for chr8, with coloring based on reported gram-staining patterns.

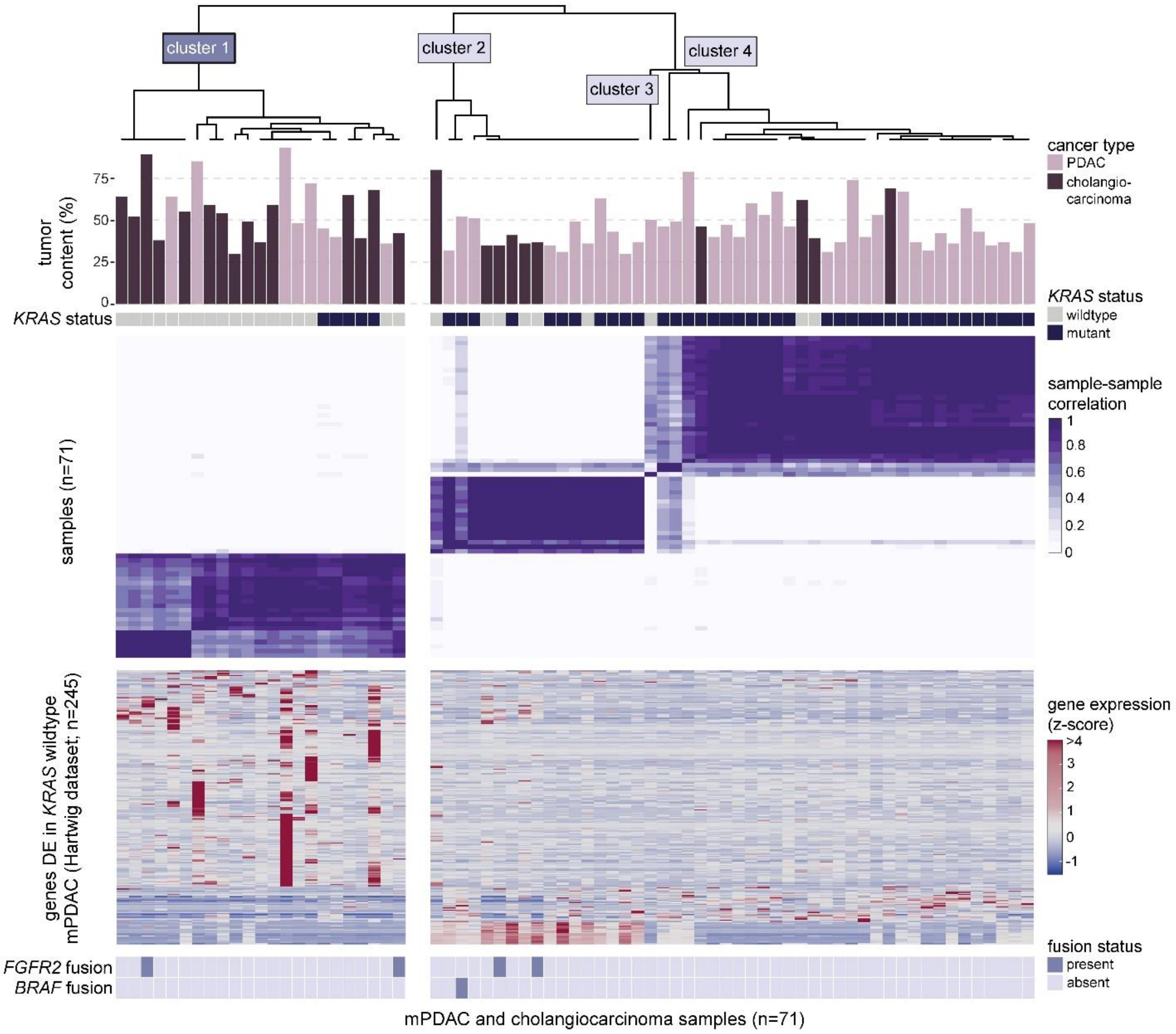

Supplementary Figure 12: Consensus clustering results of advanced PDAC and cholangiocarcinoma samples from the Hartwig validation dataset. Upper heatmap (purple/white) shows results of consensus clustering of Hartwig PDAC (n=46) and cholangiocarcinoma (n=25) samples based on mRNA expression levels (z-score) of genes found to be differentially expressed in *KRAS* wildtype PDAC samples (n=245) in the Hartwig dataset. Upper bars indicate tumor content levels for each sample, with upper grid showing *KRAS* mutation status. Lower heatmap (blue/red) shows expression patterns of the genes used for clustering. Bottom grids show gene fusion events detected in each sample.

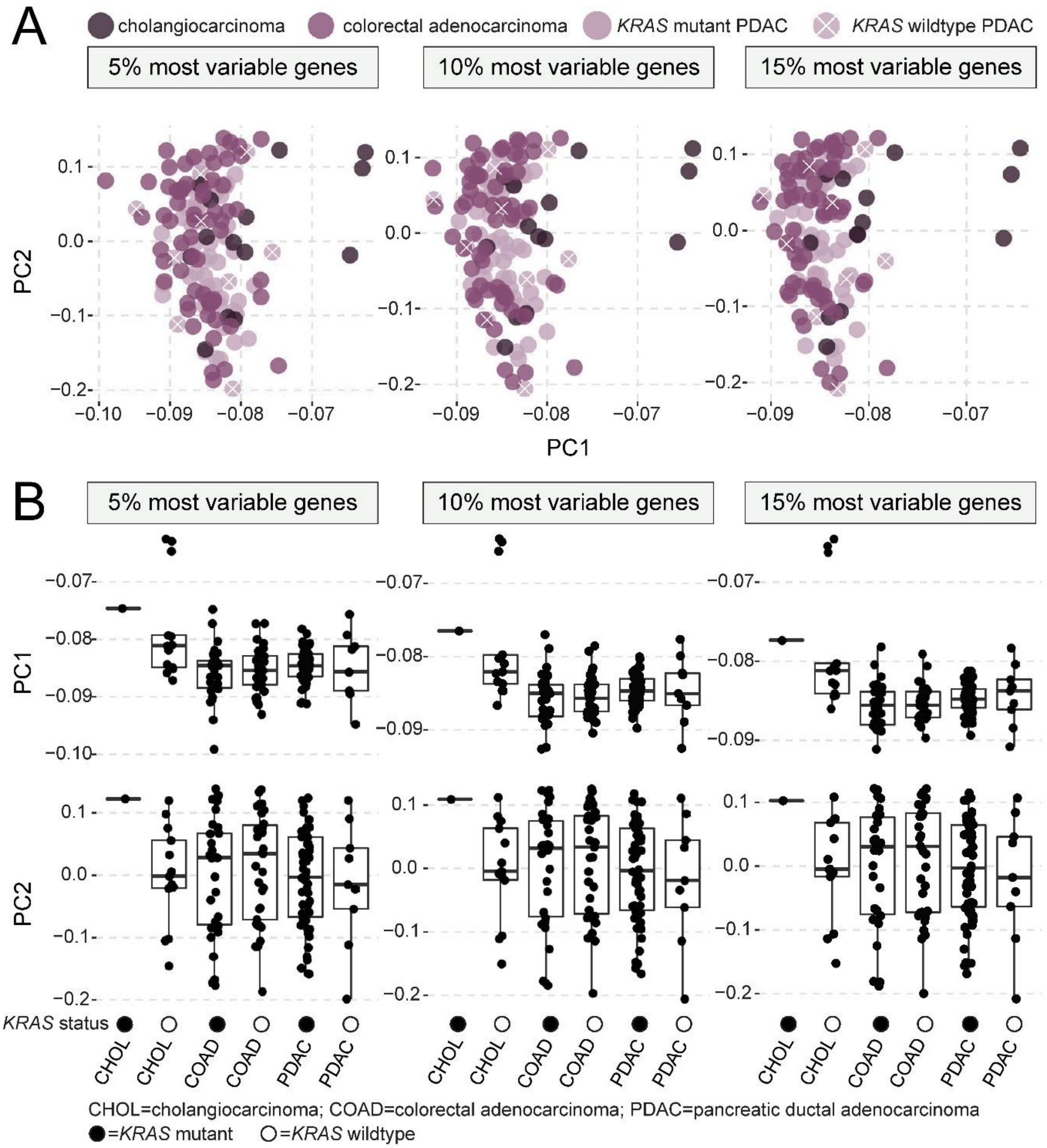

Supplementary Figure 13: **Principal component analysis of the batch-corrected pan-cancer RNA-seq dataset across variable global gene inclusion thresholds.** (A) Scatter plot showing all samples projected onto the first (x-axis) and second (y-axis) principal components (PCs) when the top 5, 10 and 15% (left to right) most variable genes (n=2,170, 4,339 and 6,509 genes) are used. *KRAS* wildtype mPDAC samples are denoted with a white cross symbol. (B) Boxplots showing PC1 (upper) and PC2 (lower) values for each sample according to cancer type and *KRAS* mutation status. Left to right: *KRAS* mutant cholangiocarcinoma (n=1), *KRAS* wildtype cholangiocarcinoma (n=13), *KRAS* mutant colorectal adenocarcinoma (n=30), *KRAS* wildtype colorectal adenocarcinoma (n=33), *KRAS* mutant PDAC (n=54), *KRAS* wildtype PDAC (n=9). Box plots indicate median (central line), 25-75% IQR (bounds of box) and whiskers extend from box bounds to the largest value no further than 1.5 times the IQR.
