# Supplementary material for: Integrative analysis of KRAS wildtype metastatic pancreatic ductal adenocarcinoma reveals mutation and expression-based similarities to cholangiocarcinoma
Source: Nat Commun. 2022 Oct 8;13:5941. doi: 10.1038/s41467-022-33718-7 (PMC9547977; doi:10.1038/s41467-022-33718-7)
Supplement: Supplementary file 2 — Description of Additional Supplementary Files [file 41467_2022_33718_MOESM2_ESM.pdf]

File Name: Supplementary Data 1

Description: Genes with significant (adjusted p<0.05) differences in copy amplification frequency between KRAS wildtype versus mutant tumors in the PanGen discovery cohort of unresectable PDAC. P values are based on two-tailed Fisher's exact tests and adjusted p values were subjected to Benjamini-Hochberg multiple test correction.

File Name: Supplementary Data 2

Description: Genes differentially expressed (adjusted p<0.05) in KRAS wildtype versus mutant tumors in the PanGen cohort of unresectable PDAC. P values based on two-tailed Wald tests, and adjusted p values were subjected to Benjamini-Hochberg multiple test correction.

File Name: Supplementary Data 3

Description: Genes conservatively differentially expressed (adjusted p<0.005, absolute log2 fold change>2.5) in KRAS wildtype versus mutant tumors in the PanGen cohort of unresectable PDAC. P values based on two-tailed Wald tests, and adjusted p values were subjected to Benjamini-Hochberg multiple test correction.

File Name: Supplementary Data 4

Description: Genes sets enriched among genes up-regulated (adjusted p<0.005, log2 fold change>2.5) in KRAS wildtype versus mutant tumors in the PanGen cohort of unresectable PDAC. P values based on one-tailed hypergeometric tests, and adjusted p values were subjected to Benjamini-Hochberg multiple test correction.

File Name: Supplementary Data 5

Description: Genes sets enriched among genes down-regulated (adjusted p<0.005, log2 fold change<-2.5) in KRAS wildtype versus mutant tumors in the PanGen cohort of unresectable PDAC. P values based on one-tailed hypergeometric tests, and adjusted p values were subjected to Benjamini-Hochberg multiple test correction.

File Name: Supplementary Data 6

Description: Proteins with unadjusted p<0.01 from results of differential protein analysis (KRAS wildtype versus mutant) in the PanGen cohort of unresectable PDAC. P values based on two-tailed loess regression, and adjusted p values were subjected to Benjamini-Hochberg multiple test correction.

File Name: Supplementary Data 7

Description: Normalized protein expression values for PROX1 and VTCN1 across the PanGen cohort of mPDAC (n=46). Values represent protein relative abundance levels on a log2 scale.
